# *AKAP1* Deficiency Attenuates Diet‐Induced Obesity and Insulin Resistance by Promoting Fatty Acid Oxidation and Thermogenesis in Brown Adipocytes

**DOI:** 10.1002/advs.202002794

**Published:** 2021-02-01

**Authors:** Lele Ji, Ya Zhao, Linjie He, Jing Zhao, Tian Gao, Fengzhou Liu, Bingchao Qi, Fei Kang, Gang Wang, Yilin Zhao, Haitao Guo, Yuanfang He, Fei Li, Qichao Huang, Jinliang Xing

**Affiliations:** ^1^ State Key Laboratory of Cancer Biology and Department of Physiology and Pathophysiology Fourth Military Medical University Xi'an Shaanxi 710032 China; ^2^ National Demonstration Center for Experimental Preclinical Medicine Education Fourth Military Medical University Xi'an Shaanxi 710032 China; ^3^ Laboratory Animal Center Fourth Military Medical University Xi'an Shaanxi 710032 China; ^4^ Department of Cardiology Xijing Hospital Fourth Military Medical University Xi'an Shaanxi 710032 China; ^5^ Department of Nuclear Medicine Xijing Hospital Fourth Military Medical University Xi'an Shaanxi 710032 China

**Keywords:** AKAP1, diet‐induced obesity, fatty acid *β*‐oxidation, insulin resistance, mitochondrial thermogenesis

## Abstract

Altering the balance between energy intake and expenditure is a major strategy for treating obesity. Nonetheless, despite the progression in antiobesity drugs on appetite suppression, therapies aimed at increasing energy expenditure are limited. Here, knockout of*AKAP1*, a signaling hub on outer mitochondrial membrane, renders mice resistant to diet‐induced obesity.*AKAP1* knockout significantly enhances energy expenditure and thermogenesis in brown adipose tissues (BATs) of obese mice. Restoring AKAP1 expression in BAT clearly reverses the beneficial antiobesity effect in *AKAP1^−/−^* mice. Mechanistically, AKAP1 remarkably decreases fatty acid β‐oxidation (FAO) by phosphorylating ACSL1 to inhibit its activity in a protein‐kinase‐A‐dependent manner and thus inhibits thermogenesis in brown adipocytes. Importantly, AKAP1 peptide inhibitor effectively alleviates diet‐induced obesity and insulin resistance. Altogether, the findings demonstrate that AKAP1 functions as a brake of FAO to promote diet‐induced obesity, which may be used as a potential therapeutic target for obesity.

## Graphical abstract

In high‐fat‐diet‐treated mice, A kinase anchoring protein 1 (AKAP1) functions as a brake molecule of fatty acid β‐oxidation by recruiting protein kinase A (PKA) and acyl‐CoA synthetase long chain family member 1 (ACSL1) to the outer mitochondrial membrane and inhibiting mitochondrial ACSL1 activity through PKA. AKAP1 elimination enhances mitochondrial ACSL1 activity and increases brown adipose thermogenesis, preventing mice from obesity.

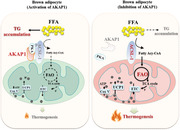

## 1 Introduction

Obesity arises from long‐term imbalance of energy, with energy intake exceeding energy expenditure, resulting in ectopic accumulation of harmful lipids in multiple tissues. Now, we know that obesity is a major risk factor for a vast number of serious medical complications, such as type 2 diabetes, fatty liver, and cardiovascular diseases, which together represent the leading causes of adult morbidity and mortality worldwide.^[^ ^1^ ^]^ Currently, obesity treatment strategies are mainly based on energy intake control, but far from enough to address this pandemic prevalence because of numerous adverse effects. In this sense, studies focusing on energy expenditure to counteract obesity are recently booming.^[^ ^2^ ^]^

Adaptive thermogenesis in brown adipose tissue (BAT) is an important contributor to energy expenditure, being crucial for energy balance and body weight maintenance. Thermogenic adipocytes burn fat to drive heat production by uncoupling‐protein‐1 (UCP1)‐dependent uncoupling of mitochondrial oxidative phosphorylation (OXPHOS) or UCP1‐independent modes, which mainly involve futile creatine and calcium cycling and/or triglyceride (TG)/free fatty acid (FFA) cycling.^[^ ^2^, ^3^, ^4^ ^]^ The thermogenic ability of BAT makes it an attractive strategy to reverse obesity. Actually, 2,4‐dinitrophenol (DNP), a chemical uncoupler of OXPHOS, has been reported to cause rapid loss of weight. However, DNP is now banned by the United States Food and Drug Administration (FDA) due to the unacceptable occurrence rate of significant adverse effects.^[^ ^5^ ^]^ In addition, most of thermogenic regulators identified in the past decades, such as targeting‐G‐protein‐coupled receptors, transient receptor potential channels, and nuclear receptors, are not ideal drug targets for obesity.^[^ ^6^ ^]^ Further studies are needed to identify key molecular regulators of BAT thermogenesis and thus develop safe and effective therapeutic options for obesity.

Mitochondria play a central role in providing an energetic basis for thermogenesis in BAT. Following lipolysis from TGs or cellular fatty acid uptake, fatty acids are converted to acyl‐CoA by acyl‐CoA synthetase (ACS) and specifically direct toward mitochondrial fatty acid β‐oxidation (FAO) via the carnitine palmitoyltransferase system. Recent studies have shown that mitochondrial FAO is crucial for active and quiescent BAT maintenance and thermogenic programing.^[^ ^7^ ^]^ However, the mechanisms underlying regulation of mitochondrial FAO are not fully understood. A kinase anchoring proteins (AKAPs) are a group of proteins with diverse structures but a common ability to bind regulatory subunit of protein kinase A (PKA), placing PKA holoenzyme into specific subcellular locations for precise function regulation.^[^ ^8^ ^]^ The mitochondria‐localized AKAP1 tethers PKA to the cytosolic surface of the outer mitochondrial membrane (OMM) in close proximity of local targets to maintain the mitochondrial function.^[^ ^9^ ^]^ A previous study has reported that AKAP1 expression is significantly downregulated in adipose tissues of obese subjects when comparing with lean subjects.^[^ ^10^ ^]^ Moreover, other studies have revealed the phenomenon that disruption of PKA regulatory subunit in mice leads to a lean phenotype.^[^ ^11^, ^12^ ^]^ These findings imply a potential role for AKAP1 in fat metabolism. However, key questions remain: does AKAP1 function in obesity development? If so, whether PKA is involved in this process and which enzyme(s) is activated by AKAP1/PKA complex? Most importantly, does AKAP1 has the potential to serve as a drug target for obesity treatment?

Here, we show that *AKAP1* knockout mice resist to high‐fat‐diet (HFD)‐induced obesity and insulin resistance. The beneficial antiobesity effect in *AKAP1^−/−^* mice can be effectively reversed by restoring the *AKAP1* expression in BAT. Furthermore, we found that *AKAP1* knockout significantly enhanced energy expenditure and mitochondrial thermogenesis in BAT of HFD mice. Interestingly, in brown adipocytes, AKAP1 functions as a brake of FAO by increasing the phosphorylation level of mitochondrial acyl‐CoA synthetase long chain family member 1 (ACSL1), to inhibit its activity in a PKA‐dependent manner. These findings suggest that AKAP1 signaling hub may create a microdomain with high PKA activity on the OMM, precisely regulating downstream FAO and mitochondrial thermogenesis. Moreover, a peptide inhibitor blocking localization of AKAP1 onto OMM also exhibited an effective antiobesity effect in HFD mice, with no significantly harmful effect on cardiac functions.

## 2 Results

### 2.1 *AKAP1* Knockout Mice are Resistant to HFD‐Induced Obesity

**Figure 1:**
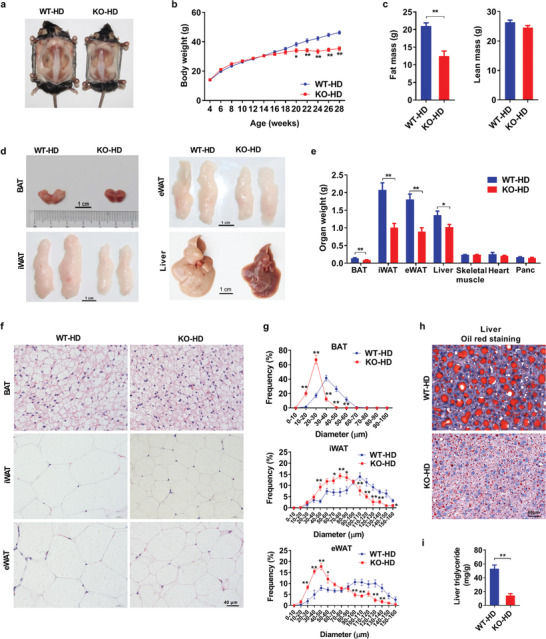
*AKAP1* knockout mice are resistant to HFD‐induced obesity. a) Representative images of WT and *AKAP1^−/−^* mice on HFD for 24 weeks. b) Body weight of WT and *AKAP1^−/−^* mice on HFD. WT‐HD: wild‐type HFD mice; KO‐HD: knockout HFD mice (*n* = 16 mice per group). c) Fat mass and lean mass of WT and *AKAP1^−/−^* mice on HFD for 24 weeks (*n* = 12 mice per group). d) Representative images of brown adipose tissue (BAT), inguinal white adipose tissue (iWAT), epididymal white adipose tissue (eWAT), and liver dissected from WT and *AKAP1^−/−^* mice on HFD for 24 weeks. Scale bar, 1 cm. e) Organ weight of WT and *AKAP1^−/−^* mice on HFD for 24 weeks (*n* = 12–13 mice per group). f) Representative H&E‐staining images of BAT, iWAT, and eWAT from WT and *AKAP1^−/−^* mice on HFD for 24 weeks. Scale bar, 40 µm. g) Distribution of adipocyte diameter (µm) in BAT, iWAT, and eWAT from WT and *AKAP1^−/−^* mice on HFD for 24 weeks (*n* = 4–6 mice per group). h) Representative oil‐red‐staining images of liver from WT and *AKAP^−/−^* mice on HFD for 24 weeks. Scale bar, 40 µm. i) Triglyceride content in liver tissue of WT and *AKAP1^−/−^* mice on HFD for 24 weeks (*n* = 6 mice per group). Data were expressed as mean ± SEM. Student's *t*‐test was used for analysis of the data in (c), (e), and (i). Two‐way ANOVA with Bonferroni's post hoc test was used for analysis of the data in (b) and (g). *p < 0.05, **p < 0.01.

To investigate the roles of AKAP1 in HFD‐induced obesity, we successfully generated *AKAP1* knockout (*AKAP1^−/−^*) mice using Clustered Regularly Interspaced Short Palindromic Repeats (CRISPR) /CRISPR‐associated protein 9 (Cas9) system (Figure S1, Supporting Information). Under normal diet (ND), *AKAP1^−/−^* mice did not show any difference in body size, weight, and fat mass when compared with wild‐type (WT) mice up to 28 weeks of age (Figure S2a–c, Supporting Information). Moreover, the weight and histology of tissues important to the regulation of nutrient metabolism were examined. No notable abnormality was observed in the BAT, inguinal and epididymal white adipose tissue (iWAT and eWAT), liver, heart, skeletal muscle, and pancreas of *AKAP1^−/−^* mice (Figure S2d–h, Supporting Information). However, with HFD for 24 weeks after weaning at 4 weeks of age, *AKAP1^−/−^* mice gained significantly less body weight (−12 g) and less body fat (−9 g) than WT mice (**Figure** **1**a–c). Consistently, the weight of major fat storage tissues including the BAT, iWAT, eWAT, and liver was all remarkably decreased in *AKAP1^−/−^* mice (Figure 1d,e). Histology analysis showed a significant decrease in adipocyte diameters in the BAT, iWAT, and eWAT of *AKAP1^−/−^* mice (Figure 1f,g). Furthermore, the lipid storage was much lower in liver of *AKAP1^−/−^* mice than in WT mice, as evidenced by reduced lipid droplets and decreased liver triglyceride content (Figure 1h,i). These data demonstrate that *AKAP1* knockout clearly attenuates HFD‐induced obesity.

### 2.2 The Improved Metabolic Profile is Observed in *AKAP1^−/−^* Mice on a HFD

**Figure 2:**
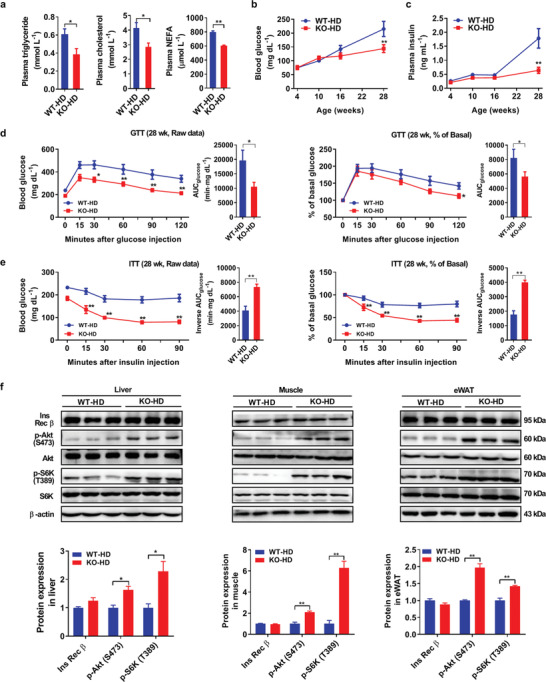
AKAP1‐deficient mice show the improved metabolic profile on a HFD. a) Plasma triglyceride, cholesterol, and nonesterified fatty acid (NEFA) levels of WT and *AKAP1^−/−^* mice on HFD for 24 weeks (*n* = 6–8 mice per group). WT‐HD: wild‐type HFD mice; KO‐HD: knockout HFD mice. b,c) Blood glucose and plasma insulin levels of WT and *AKAP1^−/−^* mice on HFD (*n* = 6 mice per group). d,e) Raw data of glucose tolerance test (GTT) and insulin tolerance test (ITT) in WT and *AKAP1^−/−^* mice on HFD for 24 weeks (left); blood glucose during GTT or ITT was expressed as a percentage of basal (right). The corresponding area under the curve (AUC) was calculated for each mouse from both groups. *n* = 7 mice per group. f) Western blotting analysis of Ins Rec β, p‐Akt (S473), Akt, p‐S6K (T389), and S6K expression in liver, muscle, and eWAT from WT and *AKAP1^−/−^* mice on a HFD for 24 weeks which were sacrificed 15 min after i.p. injection of insulin (5 U kg^−1^). β‐actin was used as a loading control (*n* = 3 mice per group). Data were expressed as mean ± SEM. Student's *t*‐test was used for analysis of the data in (a), (d) (AUC), (e) (AUC), and (f). Two‐way ANOVA with Bonferroni's post hoc test was used in (b–d) (raw data, % of basal in GTT) and (e) (raw data, % of basal in ITT). *p < 0.05, **p < 0.01.

To investigate whether the decreased lipid storage affords beneficial effects on systemic energy homeostasis in *AKAP1^−/−^* mice, the circulating metabolic profile was determined. As shown in **Figure** **2**a, the concentration of fasting plasma triglyceride, cholesterol, and nonesterified fatty acids (NEFAs) was significantly lower in *AKAP1^−/−^* mice than WT mice after 24‐week HFD. In addition, fasting blood glucose (Figure 2b) and plasma insulin (Figure 2c) were reduced in *AKAP1^−/−^* HFD mice. Glucose tolerance test (GTT) and insulin tolerance test (ITT) revealed that *AKAP1^−/−^* HFD mice exhibited a more efficient clearance of plasma glucose than WT HFD mice, as evidenced by the reduced area under the curve (AUC) of GTT and increased inverse AUC of ITT, respectively (Figure 2d,e). Furthermore, the response sensitivity of insulin was estimated in three different tissues by Western blotting analysis of insulin postreceptor signaling transduction as previously described.^[^ ^13^ ^]^ No significant expression difference of the insulin receptor *β* subunit in liver, muscle, and eWAT was observed between WT and *AKAP1^−/−^* HFD mice after injection with a supramaximal dose of insulin (5 U kg^−1^), whereas the phosphorylation of Akt (S473) and its downstream target S6K (T389) was significantly increased in three tissues of the *AKAP1^−/−^* HFD mice (Figure 2f), indicating the improved glucose and insulin tolerance in *AKAP1^−/−^* HFD mice. In comparison, no significant difference in metabolic profile and tissue insulin response was observed between WT and *AKAP1^−/−^* mice on a ND (Figure S3a–f, Supporting Information). Collectively, our data demonstrate that *AKAP1* knockout not only decreases lipid storage, but also improves circulating metabolic profile in HFD‐induced obesity, suggesting that *AKAP1^−/−^* HFD mice have greater fat utilization (i.e., fat burning).

### 2.3 *AKAP1* Knockout Enhances Energy Expenditure and BAT Mitochondrial Thermogenesis in HFD Mice

**Figure 3:**
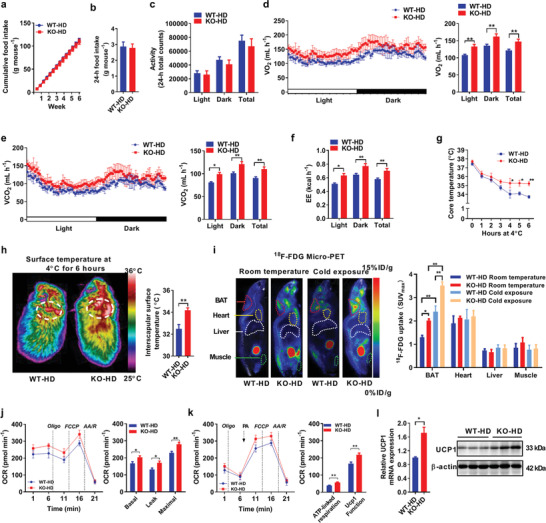
AKAP1 deficiency enhances energy expenditure and BAT activity in mice fed with a HFD. a) Cumulative food intake of WT and *AKAP1^−/−^* HFD mice was measured every 3 days from 15 to 21 weeks of age (*n* = 8–9 mice per group). b) 24 h food intake and c) physical activity of WT and *AKAP1^−/−^* HFD mice (*n* = 6 mice per group). d,e) Oxygen consumption (VO_2_) and carbon dioxide expiration (VCO_2_) were determined by metabolic cages in WT and *AKAP1^−/−^* HFD mice (*n* = 6 mice per group). f) Energy expenditure (EE) was estimated by the amount of O_2_ consumption and CO_2_ production in WT and *AKAP1^−/−^* HFD mice (*n* = 6 mice per group). g) Rectal core temperature of WT and *AKAP1^−/−^* HFD mice exposed to 4 °C for 6 h (*n* = 6 mice per group). h) Representative infrared thermography of WT and *AKAP1^−/−^* HFD mice exposed to 4 °C for 6 h (left). Surface temperature was quantified in the interscapular BAT area indicated by circle (right). *n* = 6 mice per group. i) Representative ^18^F‐FDG micro‐PET/CT images in WT and *AKAP1^−/−^* HFD mice at room temperature or cold exposure. A higher intensity of red color indicates a higher ^18^F‐FDG uptake. ID, injection dose (left). The areas of interscapular BAT, heart, liver, and muscle were marked and maximum standard uptake value (SUV_max_) was quantified (right). *n* = 6 mice per group. j) Oxygen consumption rate (OCR) of BAT mitochondria was monitored by Seahorse analyzer in WT and *AKAP1^−/−^* HFD mice (*n* = 5 mice per group). Mitochondria (5 µg) were incubated in buffer with the following compounds: 1 µM Oligomycin (Oligo), 1 µM carbonyl cyanide 4‐(trifluoromethoxy) phenylhydrazone (FCCP, F), and 2 µM each of rotenone (R) and antimycin A (AA). Basal OCR corresponds to baseline OCR minus AA/R‐insensitive OCR; leak OCR corresponds to Oligo‐insensitive OCR minus AA/R‐insensitive OCR; maximal OCR corresponds to FCCP‐induced OCR minus AA/R‐insensitive OCR. k) UCP1 function in BAT mitochondria of WT and *AKAP1^−/−^* HFD mice (*n* = 5 mice per group) was monitored by Seahorse analyzer. Respiration was measured with 1 mM GDP, 10 mM pyruvate, and 5 mM malate as substrates. Mitochondria (5 µg) were incubated in buffer with the following compounds: 1 µM Oligo, 1 µM FCCP (F), 200 µM palmitate, and 4 µM each of rotenone (R) and antimycin A (AA), respectively (*n* = 4 per group). ATP‐linked respiration corresponds to baseline OCR minus Oligo‐insensitive OCR; Uncoupling protein 1(UCP1) function corresponds to palmitate addition phase OCR minus Oligo‐insensitive OCR. l) qRT‐PCR and Western blotting analysis for UCP1 mRNA and protein expression in BAT from WT and *AKAP1^−/−^* HFD mice (*n* = 3 mice per group). Data were expressed as mean ± SEM. Student's *t*‐test was used for analysis of the data in (b), (c), (h), (j–l). One‐way ANOVA with Bonferroni's post hoc test was used for analysis of the data in (i). Two‐way ANOVA with Bonferroni's post hoc test was used in (a) and (g). The data in (d–f) were analyzed by analysis of covariance (ANCOVA) using body weight as the covariate. *p < 0.05, **p < 0.01.

To illustrate the mechanism underlying the greatly decreased fat storage in *AKAP1^−/−^* mice, the effect of *AKAP1* knockout on cumulative food intake was determined. In addition, daily food intake, physical activity, and energy expenditure (EE) were first determined in mice (18 week old) on ND and HFD using metabolic cages. Under ND or HFD feeding, no difference was observed in the food intake and physical activity between WT and *AKAP1^−/−^* mice (**Figure** **3**a–c and Figure S4a–c (Supporting Information)). However, O_2_ consumption and CO_2_ production were significantly increased in *AKAP1^−/−^* HFD mice during both the dark and light phases when compared with WT controls (Figure 3d,e). Moreover, EE estimated by the amount of O_2_ consumption and CO_2_ production during oxidation of nutrients was also significantly increased in *AKAP1^−/−^* HFD mice (Figure 3f). By contrast, no notable change was observed in O_2_ consumption, CO_2_ production, and EE between WT and *AKAP1^−/−^* mice on a ND (Figure S4d–f, Supporting Information). These data indicate that *AKAP1* knockout enhances EE only in HFD mice, which is not significantly associated with food intake or physical activity.

Given the evidence for increased EE, we then examined the cold‐induced thermogenesis. When HFD mice (28 week old) were maintained at room temperature (≈23 °C), no notable difference was observed in the core body temperature between WT and *AKAP1^−/−^* groups. When placed at 4 °C, body temperature fell progressively in WT and *AKAP1^−/−^* mice, but to a significantly greater extent in WT mice. Keeping at 4 °C for 8 h, the core body temperature of *AKAP1^−/−^* HFD mice remained significantly higher than that of WT HFD mice (Figure 3g). The superior defense of core temperature in the cold‐exposed *AKAP1^−/−^* HFD mice suggests a greater thermogenic response, especially in view of their smaller size and lesser adiposity. In agreement with these data, infrared thermographic imaging analysis revealed that *AKAP1^−/−^* HFD mice at 4 °C for 6 h had a significantly higher surface temperature than WT HFD mice, especially in the interscapular BAT area (Figure 3h). We further determined the ^18^F‐fluorodeoxyglucose (^18^F‐FDG) uptake, a reliable indication of activated BAT, using micro‐positron emission tomography (PET).^[^ ^14^, ^15^ ^]^ Our data showed that FDG uptake was higher in the interscapular BAT region of *AKAP1^−/−^* HFD mice when compared with that of WT controls during room temperature or cold exposure, whereas no difference was observed in the regions of muscle, liver, or heart between two genotypes (Figure 3i). Interestingly, *AKAP1^−/−^* ND mice also exhibited higher core body temperature and surface temperature than that in WT ND mice during cold exposure but not room temperature (Figure S4g,h, Supporting Information), indicating that knockout of *AKAP1* significantly enhanced BAT thermogenesis under high fat feeding or cold stimulation.

Furthermore, basal and maximal oxygen consumption rates (OCRs) were significantly increased in isolated BAT mitochondria of *AKAP1^−/−^* HFD mice when compared with those of WT controls (Figure 3j). Meanwhile, the function and expression of UCP1 (an indicator of mitochondrial thermogenesis) was significantly upregulated in BAT of *AKAP1^−/−^* HFD mice (Figure 3k,l). By contrast, there was no significant difference in OCR, UCP1 function and expression in BAT mitochondria between *AKAP1^−/−^* and WT ND mice (Figure S4j–l, Supporting Information). These results suggested that *AKAP1* knockout may not have significant effect on BAT thermogenesis under normal condition. However, under stressful conditions such as cold exposure or high fat feeding, *AKAP1* knockout markedly increased BAT thermogenesis and energy expenditure in mice.

### 2.4 Restoring the AKAP1 Expression in BAT Reverses the Beneficial Effect in *AKAP1^−/−^* HFD Mice

**Figure 4:**
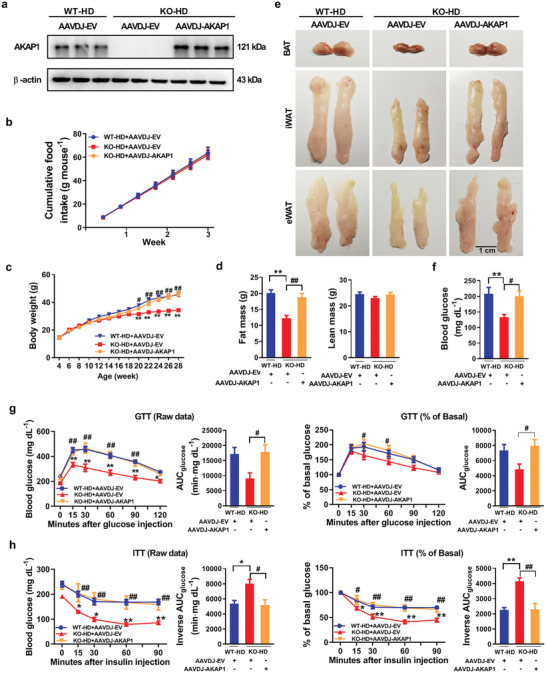
Restoring the expression of AKAP1 in BAT reverses the antiobesity effect in AKAP1‐deficient mice. a) Western blotting analysis of AKAP1 protein expression in BAT from WT and *AKAP1^−/−^* HFD mice injected with AAVDJ‐EV or AAVDJ–AKAP1 (*n* = 3 mice per group). AAVDJ‐EV, adeno‐associated virus (AAVDJ) empty vector; AAVDJ–AKAP1: AAVDJ vector expressing AKAP1. b) Cumulative food intake of WT and *AKAP1^−/−^* HFD mice injected with AAVDJ‐EV or AAVDJ–AKAP1 was measured every 3 days from 18 to 21 weeks of age (*n* = 5–6 mice per group). c) Body weight of WT and *AKAP1^−/−^* HFD mice injected with AAVDJ‐EV or AAVDJ–AKAP1 (*n* = 12 mice per group). d) Fat mass and lean mass of WT and *AKAP1^−/−^* mice on HFD injected with AAVDJ‐EV or AAVDJ–AKAP1 (*n* = 10 mice per group). e) Representative images of BAT, iWAT, and eWAT dissected from WT and *AKAP1^−/−^* HFD mice injected with AAVDJ‐EV or AAVDJ–AKAP1. Scale bar, 1 cm. f) Fasting blood glucose level of WT and *AKAP1^−/−^* HFD mice injected with AAVDJ‐EV or AAVDJ–AKAP1 (*n* = 8 mice per group). g,h) Raw data of GTT and ITT in WT and *AKAP1^−/−^* HFD mice (at 24–25 weeks of age) injected with AAVDJ‐EV or AAVDJ–AKAP1 (left); blood glucose during GTT or ITT was expressed as a percentage of basal (right). The AUC was calculated for each mouse from all groups. *n* = 5 mice per group. Data were expressed as mean ± SEM. One‐way ANOVA with Bonferroni's post hoc test was used in (d), (f), (g) (AUC), and (h) (AUC). Two‐way ANOVA with Bonferroni's post hoc test was used in (b), (c), (g) (raw data, % of basal in GTT), and (h) (raw data, % of basal in ITT). *p < 0.05, **p < 0.01, KO‐HD+AAVDJ‐EV versus WT‐HD+AAVDJ‐EV; ^#^ *p* < 0.05, ^##^ *p* < 0.01; KO‐HD+AAVDJ–*AKAP1* versus KO‐HD+AAVDJ‐EV.

To further illustrate the impact of *AKAP1* on obesity and insulin resistance, we restored AKAP1 expression in interscapular BAT of *AKAP1^−/−^* HFD mice by direct injection of adeno‐associated virus (AAVDJ) vector expressing AKAP1. As shown in **Figure** **4**a,*AKAP1* expression in BAT of *AKAP1^−/−^* HFD mice was significantly restored after intra‐BAT AAVDJ–AKAP1 injection. By contrast, AKAP1 was hardly detected in other organs, such as eWAT, iWAT, liver, muscle, and heart in *AKAP1^−/−^* HFD mice (Figure S5a, Supporting Information). Although there is no significant difference in cumulative food intake (Figure 4b), body weight and fat mass in *AKAP1^−/−^* HFD mice were significantly increased to the comparable level of WT HFD mice after restoring AKAP1 in BAT (Figure 4c,d). Consistently, following restoration of AKAP1, the size of BAT, iWAT, and eWAT was markedly increased in *AKAP1^−/−^* HFD mice and was very similar with those in WT HFD mice (Figure 4e). Furthermore, the injection of AAVDJ–AKAP1 in BAT also reversed the metabolic improvement induced by *AKAP1* knockout, which was evidenced by elevated fasting blood glucose and impaired glucose and insulin tolerance (Figure 4f–h). In order to further evaluate whether browning of white fat is involved in increased energy expenditure, UCP1 expression was determined in iWAT and eWAT of *AKAP1^−/−^* HFD mice. No typical UCP1‐positive multilocular adipocyte was observed in iWAT and eWAT of *AKAP1^−/−^* HFD mice (Figure S5b, Supporting Information). Moreover, the mRNA expression levels of BAT‐specific markers (UCP1, cell death‐inducing DNA fragmentation factor‐alpha‐like effector A (*Cidea*), deiodinase 2 (*Dio2*), PR domain containing 1(*Prdm16*), peroxisome proliferator‐activated receptor γ coactivator 1α (*Pgc1α*) and peroxisome proliferator‐activated receptor γ (*PPARγ*)) and WAT‐specific marker (adipocyte protein 2, *Ap2*) remained unchanged in iWAT and eWAT between two groups (Figure S5c,d, Supporting Information), suggesting that no obvious phenotype of WAT browning occurred in *AKAP1^−/−^* HFD mice. Thus, thermogenic phenotype should be mainly coming from BAT tissues. Our results further demonstrate a critical role of BAT AKAP1 in HFD‐induced obesity and insulin resistance.

### 2.5 AKAP1 Decreases FAO by Inhibiting *ACSL1* Activity

**Figure 5:**
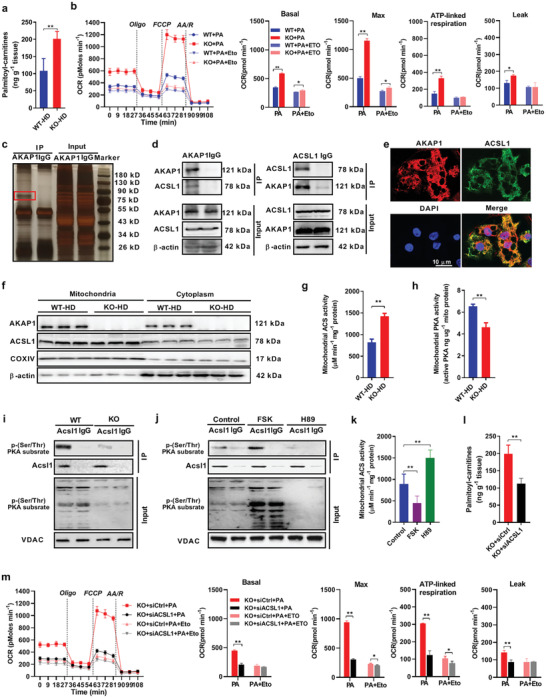
AKAP1 decreases fatty acid β‐oxidation by inhibiting ACSL1 activity. a) Palmitoyl carnitine level was measured by LC–MS/MS in BAT of WT and *AKAP1^−/−^* HFD mice (*n* = 6 mice per group). b) Fatty acid oxidation (FAO) profiles were determined by a Seahorse extracellular flux analyzer in differentiated WT and *AKAP1^−/−^* brown adipocytes after exposure to 500 µM palmitate for 24 h. Cells were treated sequentially with Oligo (3 µM), the chemical uncoupler FCCP (1 µM), R( 4 µM), and AA(4 µM) in the presence of palmitate (PA). Etomoxir (Eto, 40 µM) was used to inhibit FAO and to confirm the assay specificity. Data were from three independent experiments. c) IP assay using AKAP1 antibody or immunoglobulin G (IgG). Samples were run on Bis‐Tris Gel and then stained with Fast Silver Stain Kit. IgG, negative control antibody. d) Co‐IP assay showing interaction between AKAP1 and ACSL1 using AKAP1 antibody (left) or ACSL1 antibody (right). e) Representative images of immunofluorescence for ACSL1 (green), AKAP1–Flag (red), and nuclei (blue) in brown adipocytes infected with AAVDJ–AKAP1–Flag. Scale bar, 10 µm. f) Western blotting analysis of AKAP1 and ACSL1 expression in mitochondria or cytoplasm of BAT from WT and *AKAP1^−/−^* mice on HFD (*n* = 3 mice per group). Mitochondrial cytochrome c oxidase subunit IV (COXIV) and β‐actin were used as loading controls. g) Mitochondrial ACS activity in BAT from WT and *AKAP1^−/−^* mice on HFD (*n* = 6 mice per group). h) Mitochondrial protein kinase A (PKA) activity in BAT from WT and *AKAP1^−/−^* mice on HFD (*n* = 6 mice per group). i) The lysate of BAT mitochondria was immunoprecipitated using ACSL1 antibody and blotted with an anti‐phospho‐(Ser/Thr) PKA substrate antibody in WT and *AKAP1^−/−^* mice on HFD. Voltage‐dependent anion channels (VDAC) was used as a loading control. j) The lysate of BAT mitochondria was immunoprecipitated using ACSL1 antibody and blotted with anti‐phospho‐(Ser/Thr) PKA substrate antibody in HFD mice treated with PKA activator forskolin (FSK) or PKA inhibitor H89. 50 µL of FSK (80 µM) or H89 (10 µM) was injected into interscapular BAT. BAT samples were collected 2 h after injection. VDAC was used as a loading control. k) Mitochondrial ACS activity in BAT from mice treated with PKA activator (FSK, 80 µM) or PKA inhibitor (H89, 10 µM). *n* = 6 mice per group. l) Palmitoyl carnitine levels were measured by LC–MS/MS in siRNA‐injected BAT of *AKAP1^−/−^* mice (*n* = 6 mice per group). siACSL1: siRNA against ACSL1; siCtrl: control siRNA. m) Fatty acid oxidation (FAO) profiles in *AKAP1^−/−^* brown adipocytes with siRNA transfection were determined by a Seahorse extracellular flux analyzer. Data were from three independent experiments. Data were expressed as mean ± SEM. Student's *t*‐test was used in (a), (b), (g), (h), (l), and (m). One‐way ANOVA with Bonferroni's post hoc test was used in (k). *p < 0.05, **p < 0.01.

Considering that fatty acids serve as the main fuel for BAT thermogenesis, we then determined whether AKAP1 inhibited BAT thermogenesis via regulating fatty acids metabolism. As shown in **Figure** **5**a, *AKAP1* knockout significantly increased palmitoyl carnitine (a substrate for FAO) level in BAT of HFD mice. Increased palmitoyl carnitine level may be associated with enhanced FAO or reduced FFA utilization. To interpret this change, we further evaluated FAO in brown adipocytes using the Seahorse analyzer in the presence of the exogenous palmitate (Figure 5b). Our data showed that *AKAP1* knockout (KO) significantly enhanced both basal and maximal oxygen consumption in brown adipocytes. These effects were blocked by the treatment of etomoxir, a carnitine palmitoyltransferase 1 (CPT1) inhibitor, confirming that the increased oxygen consumption was indeed derived from the oxidation of exogenous fatty acids. Moreover, both adenosine triphosphate (ATP)‐linked respiration and proton leak respiration were increased in *AKAP1* KO brown adipocytes under palmitate (PA) treatment when compared with WT brown adipocytes, implying that both UCP1‐dependent and ‐independent thermogenesis were involved. To elucidate the molecular mechanism underlying the decreased FAO by AKAP1, the proteins physically interacting with AKAP1 were identified by immunoprecipitation–mass spectrometry (IP–MS) technology. As shown in Figure 5c, an obvious band around 80 kDa was observed in product immunoprecipitated by anti‐*AKAP1* antibody. Long chain fatty acid–CoA ligase 1 (ACSL1), which is located on the OMM and directs fatty acids into mitochondria for β‐oxidation,^[^ ^16^, ^17^ ^]^ was MS‐identified among top AKAP1‐interacting proteins. The physical interaction and mitochondrial colocation between AKAP1 and ACSL1 were further confirmed in mouse brown adipocytes treated with palmitate for 24 h via co‐IP and immunofluorescence assays (Figure 5d,e). We further investigated whether AKAP1 affected the biological function of ACSL1. Notably, *AKAP1* knockout had no effect on the expression level and subcellular location of ACSL1 (Figure 5f and Figure S6a (Supporting Information)). However, the enzyme activity of mitochondrial ACS, 90% of which is composed of ACSL1 activity in adipocytes as previously described,^[^ ^18^ ^]^ was significantly increased in BAT from *AKAP1^−/−^* HFD mice (Figure 5g). Given that*AKAP1* functions to tether PKA onto mitochondria, it is intriguing to speculate that AKAP1/PKA may phosphorylate mitochondrial ACSL1 and thus regulate its activity. As shown in Figure 5h, the enzyme activity of mitochondrial PKA was significantly reduced in BAT from *AKAP1^−/−^* HFD mice. By contrast, there was no significant difference of total PKA activity in BAT between wild‐type and *AKAP1^−/−^* HFD mice (Figure S6b, Supporting Information). We next sought to demonstrate that ACSL1 is indeed a phosphorylation target of PKA. Due to lack of data about the phosphorylation sites in ACSL1, ACSL1 was immunoprecipitated from mitochondria and its phosphorylation level was analyzed by immunoblotting with anti‐phospho‐(Ser/Thr) PKA substrate antibody. Consistently, the level of mitochondrial ACSL1 phosphorylation (p‐ACSL1) was decreased in BAT from *AKAP1^−/−^* HFD mice when compared with that from WT controls (Figure 5i). Moreover, mitochondrial p‐**ACSL1** in mouse BAT was strongly increased and reduced by the PKA activator forskolin (FSK) and inhibitor H89, respectively (Figure 5j and Figure S6c (Supporting Information)). By contrast, the opposite effect of FSK and H89 on the enzyme activity of mitochondrial ACS was observed, indicating an inverse relationship between the enzyme activity and mitochondrial p‐ACSL1 (Figure 5k). To further confirm the role of ACSL1 in AKAP1‐regulated FAO, the expression of ACSL1 was downregulated in primary brown adipocytes by *ACSL1* small interfering RNA (siRNA) (Figure S6d, Supporting Information). As shown in Figure S6e (Supporting Information) and Figure 5l,m,*ACSL1* knockdown significantly decreased mitochondrial ACS activity, palmitoyl carnitine level, and FAO in *AKAP1^−/−^* brown adipocytes incubated with palmitate. Furthermore, the abundance of ACSL1 in mouse BAT was much higher than that in WAT (Figure S6f, Supporting Information), suggesting that AKAP1–PKA–ACSL1 signaling pathway may play a major role in BAT fatty acid metabolism but not in WAT. Together, our findings suggest that AKAP1 acts as a brake of FAO by inhibiting mitochondrial ACSL1 activity in BAT.

### 2.6 *A* *KAP1* Knockout Increases Mitochondrial Thermogenesis in Brown Adipocytes by Enhancing FAO

**Figure 6:**
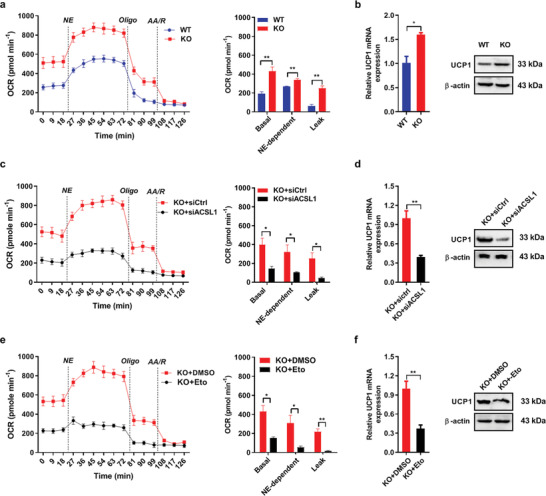
AKAP1 deficiency increases mitochondrial thermogenesis in brown adipocyte by enhancing fatty acid β‐oxidation and subsequent UCP1 upregulation. a) OCR measurement in differentiated WT and *AKAP1^−/−^* brown adipocytes pretreated with 500 µM palmitate for 24 h. During measurement, brown adipocytes were sequentially stimulated with the following compounds: 1 µM Norepinephrine (NE), 3 µM Oligo, and 4 µM each of AA/R. Basal OCR corresponds to baseline OCR minus AA/R‐insensitive OCR; NE‐dependent OCR corresponds to NE addition phase OCR minus Basal OCR. Leak OCR corresponds to Oligo‐insensitive OCR minus AA/R‐insensitive OCR. Data were from three independent experiments. b) UCP1 mRNA and protein expression in differentiated WT and *AKAP1^−/−^* brown adipocytes pretreated with 500µM palmitate for 24 h. Data were from three independent experiments. c) OCR measurement in differentiated *AKAP1^−/−^* brown adipocytes with siRNA transfection. Data were from three independent experiments. d) UCP1 mRNA and protein expression in differentiated *AKAP1^−/−^* brown adipocytes with siRNA transfection. Data were from three independent experiments. e) OCR measurement in differentiated WT and *AKAP1^−/−^* brown adipocytes with Eto treatment. Dimethyl sulfoxide (DMSO) was used as control. Data were from three independent experiments. f) UCP1 mRNA and protein expression in differentiated *AKAP1^−/−^* brown adipocytes with Eto treatment. Data were from three independent experiments. Data were expressed as mean ± SEM. Student's *t*‐test was used in (a–f). *p < 0.05, **p < 0.01.

Next, we further verified the critical role of AKAP1‐mediated FAO in mitochondrial thermogenesis of brown adipocytes. Primary precursor adipocytes were isolated from BAT of *AKAP1^−/−^* mice and differentiated and then the OCR and UCP1 expressions were evaluated under different treatments after culturing with 500 µM palmitate for 24 h. Basal, norepinephrine (NE)‐dependent and leak OCR were significantly increased in *AKAP1^−/−^* brown adipocytes when compared with WT controls (**Figure** **6**a). Additionally, UCP1 mRNA and protein levels were also significantly upregulated in *AKAP1^−/−^* brown adipocytes (Figure 6b). These data demonstrated the increased mitochondrial thermogenesis by *AKAP1* knockout in brown adipocytes. Moreover, mitochondrial long chain fatty acid β‐oxidation was inhibited by ACSL1 knockdown and etomoxir, a pharmacological inhibitor of CPT1. As shown in Figure 6c–f, ACSL1 knockdown and etomoxir treatment significantly decreased OCR and UCP1 expression in *AKAP1^−/−^* brown adipocytes, indicating the critical role of FAO in mitochondrial thermogenesis. Collectively, all these findings indicate that *AKAP1* knockout enhances FAO to increase mitochondrial thermogenesis in brown adipocytes.

### 2.7 AKAP1 is Feedback Downregulated under Obese Conditions

**Figure 7:**
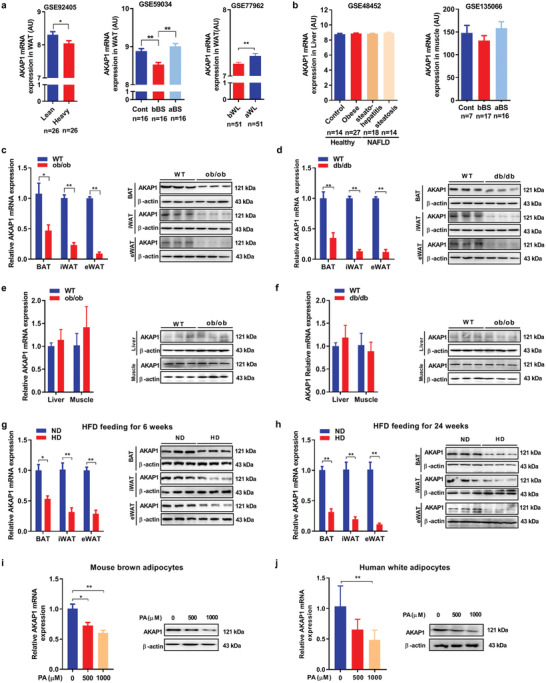
AKAP1 is feedback downregulated under obese conditions. a) The mRNA expression levels of AKAP1 were evaluated in the human adipose tissue based on public microarray expression datasets (GSE92405, GSE59034, GSE77962). Cont, never‐obese control; bBS, before bariatric surgery; aBS, after bariatric surgery; bWL, before weight loss (at study start); aWL, after weight loss period. b) The mRNA expression levels of AKAP1 were evaluated in the human liver and muscle based on public microarray expression datasets (GSE48452, GSE135066). c,d) Relative mRNA expression (left) and Western blotting analysis (right) of AKAP1 expression in adipose tissues from ob/ob and db/db mice (*n* = 3 mice per group). e,f) Relative mRNA expression (left) and Western blotting analysis (right) of AKAP1 expression in liver and muscle from ob/ob and db/db mice (*n* = 3 mice per group). g,h) Relative mRNA expression (left) and Western blotting analysis (right) of AKAP1 expression in adipose tissues from mice after HFD feeding for 6 and 24 weeks (*n* = 3 mice per group). i) Relative mRNA expression (left) and Western blotting analysis (right) of AKAP1 in mouse brown adipocytes pretreated with palmitate for 24 h. Data were from three independent experiments. j) Relative mRNA expression (left) and Western blotting analysis (right) of AKAP1 in human adipocytes pretreated with palmitate for 24 h. Data were from at least three independent experiments. Data were expressed as mean ± SEM. Student's *t*‐test was used in (a) (GSE92405, GSE77962) and (c–h). One‐way ANOVA with Bonferroni's post hoc test was used in (a) (GSE59034), (b), (i), and (j). *p < 0.05, **p < 0.01.

We further investigated the association of AKAP1 expression and obese conditions in human and mice. First, we evaluated the mRNA expression levels of AKAP1 in obese and lean human subjects using public microarray datasets from Gene Expression Omnibus (GEO) database (**Figure** **7**a). Interestingly, AKAP1 mRNA level was significantly decreased in subcutaneous adipose tissues (SATs) from the heavy one of twin pairs when compared with the lean one. Moreover, when compared with that of health controls, AKAP1 mRNA level was significantly downregulated in SAT of patients with obesity, which was effectively reversed by bariatric surgery. In addition, AKAP1 expression was upregulated after low‐calorie diet‐induced weight loss in overweight and obese humans. However, no significant change of AKAP1 expression was observed in other metabolism‐associated tissues including liver and muscle among different metabolic conditions (Figure 7b). Next, the mRNA and protein expressions of AKAP1 were determined in different tissues of mice under obese conditions. As shown in Figure 7c,d, AKAP1 mRNA and protein expressions were significantly downregulated in adipose tissues including BAT, eWAT, and iWAT from ob/ob and db/db mice when compared with those from control group. By contrast, there was no significant difference of AKAP1 expression in liver and muscle between control and ob/ob or db/db mice (Figure 7e,f). In addition, the effect of HFD on AKAP1 expression was evaluated in adipose tissues of mice after feeding for 6 and 24 weeks. As shown in Figure 7g,h, AKAP1 expression was significantly downregulated in BAT, eWAT, and iWAT in a time‐dependent manner. Finally, HFD‐induced downregulation of AKAP1 was further confirmed in primary mouse brown adipocytes and human white adipocytes through treatment with 500 or 1000 µM palmitate (Figure 7i,j). Collectively, these findings demonstrate that AKAP1 expression is progressively downregulated under obese condition, which suggests a negative feedback regulation of AKAP1 expression to fight against its obese‐promoting effect.

We then further investigated the mechanism underlying the downregulation of AKAP1 in obesity. Potential transcription factor SP1 (also known as specificity protein 1) for AKAP1 was predicted by searching PROMO, Jaspar program, and hTFtarget database (Figure S7a, Supporting Information). Then, several putative SP1 binding sites were further identified in the 5′‐flanking region of human AKAP1 by searching the hTFtarget database (http://bioinfo.life.hust.edu.cn/hTFtarget/#!/) (Figure S7b, Supporting Information), which are conserved between human and mouse. Next, we evaluated the mRNA expression levels of SP1 in obese and lean human subjects using public microarray datasets from GEO database. Scatter plot analysis showed a positive correlation between the mRNA levels of AKAP1 and SP1 in subcutaneous adipose tissues from health and obese subjects (Figure S7c, Supporting Information). SP1 mRNA level was also significantly downregulated in adipose tissue of overweight and obese humans, which was consistent with the downregulation of AKAP1 in obesity (Figure S7d, Supporting Information). Moreover, the decreased *SP1* protein expression was observed in BAT from HFD mice (Figure S7e, Supporting Information). Chromatin immunoprecipitation (ChIP) assay demonstrated that SP1 directly bound to the promoter region of AKAP1 gene in mouse BAT tissues (Figure S7f, Supporting Information). Moreover, Western blotting analysis revealed that SP1 knockdown significantly decreased the expression of AKAP1 and exacerbated the downregulation of AKAP1 induced by high concentrations of palmitate treatment (Figure S7g, Supporting Information). By contrast, SP1 overexpression exhibited an opposite effect (Figure S7h, Supporting Information). Taken together, these results suggested that downregulated AKAP1 in the obese human and mouse adipose tissue was at least in part due to the decreased expression of transcriptional factor SP1.

### 2.8 AKAP1 Inhibitor Alleviates HFD‐Induced Obesity and Insulin Resistance

In order to test whether AKAP1 has the potential to serve as a target for obesity treatment, two peptides spanning full length (30 aa) or core region (21 aa) of the mitochondrial targeting domain in AKAP1, which were, respectively, named as AP‐30 and AP‐21, were synthetized and used as an competitive inhibitor to block localization of AKAP1 onto the OMM as previously described.^[^ ^19^ ^]^ We first preliminarily investigated the effect of two peptides on HFD‐induced obesity by a short‐term treatment (daily subcutaneous injection into interscapular BAT area for 7 days). Our data showed that no significant difference of body weight was observed between control and AP‐30‐treated mice at different concentrations. By contrast, AP‐21‐treated mice showed a trend of body weight loss in a dose‐dependent manner (Figure S8a, Supporting Information). After that, a mutant AP‐21, which lost the ability to target mitochondria,^[^ ^20^ ^]^ was synthetized to serve as a control peptide. As expected, the treatment with AP‐21 peptide significantly inhibited AKAP1 mitochondria localization. By contrast, the mutant peptide did not remarkably affect AKAP1 mitochondria localization (Figure S8b, Supporting Information). Therefore, the dose of 2 mg kg^−1^ was used to further investigate the antiobesity effect of AP‐21 with longer treatment duration (4 weeks) in early and late phases of HFD‐induced obesity.

**Figure 8:**
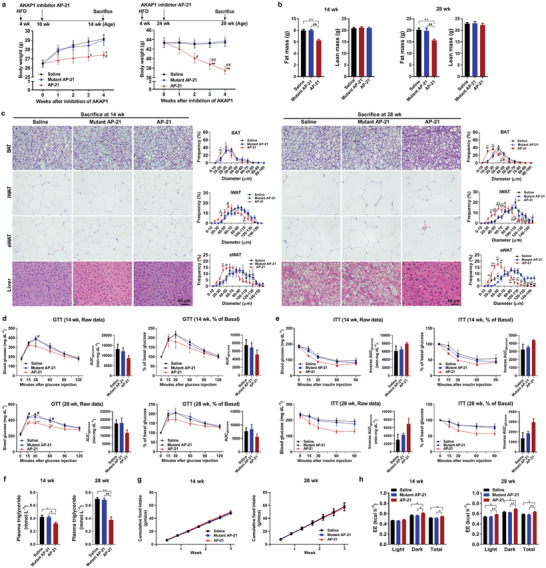
AKAP1 inhibitor effectively alleviates diet‐induced obesity and insulin resistance. a) Body weight of HFD‐treated mice injected with *AKAP1* inhibitor AP‐21, saline, or mutant AP‐21 (*n* = 10–12 mice per group). b) Fat mass and lean mass of HFD‐treated mice injected with AKAP1 inhibitor, saline, or mutant AP‐21 (*n* = 8–10 mice per group). c) Representative H&E‐stained sections of BAT, iWAT, eWAT, and liver dissected from HFD mice treated with saline, mutant AP‐21, or AP‐21 at early and late phases (left). Scale bar, 40 µm. Distribution of adipocyte diameter (μm) in BAT, iWAT, and eWAT from HFD mice treated with saline or AP‐21 at early and late phases (right). *n* = 6–9 mice per group. d,e) Raw data of GTT and ITT in HFD‐treated mice injected with AKAP1 inhibitor (left); blood glucose during GTT or ITT was expressed as a percentage of basal (right). The AUC was calculated for each mouse from all groups. *n* = 5 mice per group. f) Plasma triglyceride level of HFD‐treated mice injected with saline or AP‐21 at early and late phases (*n* = 5–6 mice per group). g) Cumulative food intake of HFD mice with treatments as indicated was measured every 3 days from 10 to 13 weeks of age and 24 to 27 weeks of age, respectively (*n* = 5–8 mice per group). h) EE was estimated by the amount of O_2_ consumption and CO_2_ production in HFD‐treated mice injected with AKAP1 inhibitor (*n* = 4 mice per group). Data were expressed as mean ± SEM. Two‐way ANOVA with Bonferroni's post hoc test was used in (a), (c), (d) (raw data, % of basal in GTT), (e) (raw data, % of basal in ITT), and (g). One‐way ANOVA with Bonferroni's post hoc test was used in (b), (d) (AUC), (e) (AUC), and (f). ANCOVA with body weight as the covariate was used in (h). *p < 0.05, **p < 0.01, AP‐21 versus saline; ^#^ *p* < 0.05, ^##^ *p* < 0.01, AP‐21 versus mutant AP‐21.

As shown in **Figure** **8**a,b, the body weight and fat mass of HFD mice were significantly decreased by AP‐21 long‐term treatment (4 weeks) beginning at both early and late phases of obesity (HFD feeding for 6 and 20 weeks, respectively) when compared with mutant AP‐21 or saline treatment. Histology analysis showed a significant decrease in adipocyte diameters in the BAT, iWAT, and eWAT and an obvious reduction of hepatic steatosis in mice with AP‐21 treatment (Figure 8c). Furthermore, AP‐21 treatment also showed the metabolic benefits, as evidenced by decreased fasting plasma triglyceride (Figure 8f). Besides, AP‐21‐treated mice had a trend toward improved glucose intolerance and insulin tolerance (Figure 8d,e). As is shown in Figure 8g, there was no significant difference in cumulative food intake among three groups, whereas AP‐21 treatment significantly enhanced EE in HFD mice (Figure 8h). In addition, the side effects of AP‐21 on other organs were also evaluated. Histological analysis of kidney, heart, and muscle showed no remarkable change in the morphology after AP‐21 treatment (Figure S8c, Supporting Information). The functions of kidney, liver, and heart were further determined. As shown in Figure S8d (Supporting Information), there were no significant changes for the level of blood urea nitrogen (BUN), serum creatinine (Cre), alanine aminotransferase (ALT), and aspartate aminotransferase (AST) after AP‐21 treatment. Similarly, echocardiography analysis also showed that AP‐21 had no significant detrimental effects on cardiac function in mice (Figure S8e, Supporting Information). Collectively, our results indicated that *AKAP1* inhibitor (2 mg kg^−1^ per day) strongly reduced HFD‐induced obesity and insulin resistance and had no significant adverse effects on the function of other organs.

## 3 Discussion

Despite intensive lifestyle modifications, the disease severity of obesity warrants further aggressive intervention. Currently, six major FDA‐approved drugs have been used for antiobesity, which can be classified into two types, anorectics to suppress appetite and the pancreatic lipase inhibitor to reduce intestinal fat absorption. Unfortunately, most of them have undesirable adverse effects.^[^ ^6^, ^21^ ^]^ Considering critical roles of thermogenic brown adipocytes in energy balance, the intervention to increase “brown” fat mass or activity is becoming an attractive strategy for prevention and treatment of obesity. In the present study, we for the first time demonstrated that AKAP1 played a critical role in HFD‐induced obesity and systemic insulin resistance. Our data showed that lack of AKAP1 signaling significantly alleviated HFD‐induced adiposity, decreased ectopic fat accumulation in liver, improved blood metabolic parameters and insulin sensitivity, indicating AKAP1 as a driving force for diet‐induced obesity and metabolic dysfunctions. Furthermore, a competitive peptide inhibitor blocking the localization of AKAP1 onto OMM clearly afforded an antiobesity effect in HFD mice. Our findings strongly suggest that AKAP1 may be used as a new target molecule for a potential therapeutic strategy of obesity.

BAT, which is specialized for energy expenditure, plays a central role in the maintenance of energy homeostasis through adaptive thermogenesis. In adult human, BAT is located mainly in supraclavicular, cervical, paravertebral, and perirenal depots, contributes to metabolic homeostasis, and may protect against obesity.^[^ ^15^, ^22^, ^23^ ^]^ Thus, increasing BAT thermogenesis is becoming a viable strategy to treat obesity and understanding the critical mechanisms that drive BAT activity now has great clinical implications. In the present study, our results revealed that AKAP1 deficiency effectively enhanced BAT activity and whole‐body energy expenditure, protecting mice against obesity and related metabolic disorders. Importantly, restoration of AKAP1 specifically in the BAT of *AKAP1^−/−^* mice dramatically increased overall body weight and induced glucose and insulin intolerance, indicating that BAT AKAP1 expression plays a key role in HFD‐induced obesity and whole‐body metabolic dysfunction.

Mechanistically, AKAP1 acts as a brake to inhibit mitochondrial FAO, which is a critical regulator in BAT thermogenesis. A previous study has showed that CPT1 (a rate‐limiting enzyme in FAO) activity and FAO is decreased in BAT of diabetic rats.^[^ ^24^ ^]^ Moreover, enhancing FAO by CPT1 overexpression significantly decreases TG content and improves insulin sensitivity under obese conditions.^[^ ^25^, ^26^, ^27^ ^]^ These findings suggest that enhancing FAO may be an appealing strategy to increase energy expenditure and fight against obesity‐induced metabolic disorders. Here, we found that *AKAP1* knockout notably increased palmitoyl carnitine level and mitochondrial FAO in brown adipocytes. Along this line, *AKAP1* knockout enhanced BAT thermogenesis, as evidenced by elevated OCR and upregulated thermogenic gene UCP1. Interestingly, our results further showed that ATP‐linked respiration was significantly increased in *AKAP1^−/−^* brown adipocytes when compared to WT brown adipocytes. Similar results were also obtained in *AKAP1^−/−^* BAT mitochondria. Considering the upregulated UCP1 activity in *AKAP1^−/−^* mitochondria and the proton leak respiration in *AKAP1^−/−^* brown adipocytes, it is reasonable to believe that both UCP1‐dependent and ‐independent thermogenesis was involved in *AKAP1* KO‐mediated thermogenesis. Furthermore, CPT1 inhibitor etomoxir significantly blocked these effects. Collectively, our data demonstrate mitochondrial FAO and subsequent thermogenesis as a novel link between BAT AKAP1 and whole‐body energy expenditure.

AKAP1 binds to both RI and RII regulatory subunits of PKA and orchestrates the spatial organization and temporal regulation of PKA signaling. Previous studies have demonstrated that PKA RIIβ deficiency enhances energy expenditure and protects mice from obesity, insulin resistance, and dyslipidemia.^[^ ^12^, ^28^ ^]^ However, PKA may not be an ideal drug target for obesity since whole‐body knockout of RIIβ has markedly diminished WAT despite normal food intake, which often causes serious health problem under certain circumstances.^[^ ^11^ ^]^ Interestingly, our data showed that the knockout of *AKAP1*, the scaffold protein that anchor PKA onto mitochondria, resulted in a different lean phenotype when compared to RII*β* knockout mice. We found that *AKAP1* knockout only induced BAT thermogenesis and weight loss on HFD, but not on ND. Furthermore, our data demonstrated that *AKAP1* knockout inhibited mitochondrial PKA activity but had no effect on total PKA activity in BAT. These results further confirmed the concept that different AKAPs confer the precise intracellular locations of PKA and specially regulate PKA‐dependent substrate phosphorylation at local site.^[^ ^29^ ^]^ Thus, inhibition of AKAP1 or its anchored PKA, but not total PKA, may have a large potential in fighting obesity with higher safety.

More importantly, in the present study, no notable abnormality was observed in the adipose tissue, liver, heart, skeletal muscle, and pancreas of *AKAP1^−/−^* mice, and systemic metabolism remains unchanged in *AKAP1^−/−^* mice on a ND. Specifically, AKAP1 inhibitor treatment has no significant detrimental effects on cardiac function in HFD mice. Consistently, previous studies have also reported that AKAP1 deficiency did not affect cardiovascular and renal functions under basal conditions.^[^ ^30^, ^31^, ^32^ ^]^ In comparison, in mice subjected to different disease models, AKAP1 deficiency exacerbates cardiovascular, lung, and cerebral vulnerability.^[^ ^30^, ^31^, ^32^, ^33^, ^34^, ^35^ ^]^ These findings suggest that AKAP1 could be a safe therapeutic target for the treatment of obesity under specific physiological conditions. Further studies are needed to evaluate the safety of targeting BAT AKAP1. New approaches targeting BAT AKAP1 are encouraged to limit the potential adverse effects.

In the present study, ACSL1 has been identified as a new target for*AKAP1*/PKA signaling complex. ACSL1 mediates the conversion of fatty acids to acyl‐CoA and specifically directs them toward mitochondrial FAO.^[^ ^16^ ^]^ In highly oxidative tissues including BAT, ACSL1 constitutes 90% of total ACS activity and its absence severely diminishes FAO, although the remaining ACSL isoforms are sufficient for normal TG and membrane phospholipid biosynthesis.^[^ ^16^, ^18^, ^36^ ^]^ A previous study has indicated that ACSL1 is highly modified post‐translationally and these modifications can be expected to alter enzymatic function.^[^ ^37^ ^]^ Specifically, an early study has reported that enzyme activity of ACSL1 is reduced in rat adipocytes after incubation with the membrane‐permeable cyclic adenosine monophosphate (cAMP) analog, dibutyryl‐cAMP.^[^ ^38^ ^]^ For the first time, our data indicated that AKAP1 interacted with ACSL1 on the OMM. Although *AKAP1* knockout did not affect mitochondrial location of ACSL1, it significantly decreased the phosphorylation level of ACSL1 and enhanced mitochondrial ACS activity in a PKA‐dependent manner in brown adipocytes. Collectively, our data indicate that AKAP1 recruits PKA onto the OMM to form a signaling microdomain with high PKA activity. Meanwhile, AKAP1 also recruits ACSL1 into this microenvironment, resulting in a higher phosphorylation level of ACSL1 and a lower ACSL1 activity around the surface of the OMM, which inhibits FAO and mitochondrial thermogenesis in BAT.

In contrast to AKAP1, which is ubiquitously expressed in many tissues, ACSL1 has been reported to be abundant in adipose tissue, liver, and heart.^[^ ^39^ ^]^ As previously reported, ACSL1 contributes to 80% of total ACSL activity in adipose tissue. FAO rates in BAT mitochondria of *ACSL1^−/−^* mice were 60% lower than of control mice.^[^ ^16^ ^]^ In the present study, we further found that the abundance of ACSL1 in mouse BAT was much higher than that in WAT. Since FAO is not a major metabolic pathway in WAT, it is reasonable to speculate that ACSL1 may not play an important role in WAT fatty acid metabolism. By contrast, liver tissues also have other major ACSL isoforms, such as ACSL4 and ACSL5. Eliminating ACSL1 in liver resulted in only 25–35% decrease in long chain acyl‐CoA content.^[^ ^39^ ^]^ In this sense, we believe that AKAP1–PKA–ACSL1 signaling pathway may be specifically conserved in only a few organs.

Growing evidences indicate that humans are predisposed to conserve body fat as a factor of survival.^[^ ^40^ ^]^ Here, we demonstrate that mitochondrial AKAP1 functions as a novel “thrifty” gene for fat storage. As expected, our data indicated that AKAP1 expression was feedback downregulated in adipose tissues under obese condition, which is highly consistent with a previous study.^[^ ^10^ ^]^ In addition, high concentrations of palmitate markedly downregulated AKAP1 mRNA and protein expression levels in human and mouse adipocytes. These data suggest a negative feedback regulation of AKAP1 expression as a physiological mechanism to prevent obesity.

## 4 Conclusion

Overall, our study highlights *AKAP1* as a novel brake molecule of mitochondrial FAO in the setting of energy excess, emphasizing the importance of AKAP1‐mediated BAT thermogenesis in antiobesity. We provide a mechanism to explain the critical role of *AKAP1* in FAO and BAT thermogenesis. Our work preliminarily verified the antiobesity effect of AKAP1 peptide inhibitor, thus paving the way for developing a new strategy targeting AKAP1 for antiobesity therapies while maintaining the healthy regulation under normal diet.

## 5 Experimental Section

##### Mice Care

All mice were maintained in groups of 3–5 under a 12 h light–dark cycle with a room temperature (22 ± 0.5 °C) (if not specified) in a specific pathogen free animal center at Fourth Military Medical University. Mice had free access to water and food unless otherwise stated. Beginning at weaning (4 weeks of age), male mice were divided into ND (10% calories from fat) and HFD (60% calories from fat) groups. All animal experiments were conducted according to the guidelines of the Institutional Animal Care and Use Committee of the Fourth Military Medical University (Permission Number: IACUC‐20170104).

##### Generation of Genetically Modified Mice

*AKAP1^−/−^* mice on a C57BL6/N background were generated using CRISPR–Cas9 method. The detailed process is available in the Supporting Information.

##### Primary Culture and Differentiation of Human Adipose‐Derived Stromal Cells

Human abdominal lipoaspirate tissues were obtained from three healthy male individuals. Human adipose‐derived stromal cells (hASCs) were isolated as previously described.^[^ ^41^ ^]^ Briefly, abdominal lipoaspirate tissues were cleaned and digested in 0.075% collagenase I (17100017, Thermo Fisher Scientific). Cells were cultured in growth medium (Dulbecco's Modified Eagle Medium (DMEM)/F12, C11330500BT, Gibco medium plus 10% fetal bovine serum (FBS), 100 I.U. penicillin, and 100 µg mL^−1^ streptomycin) for 24 h in a CO_2_ incubator (37 °C, 5% CO_2_). The medium was changed every 3 days. To induce differentiation of hASCs into mature adipocytes, cells were cultured in DMEM/F12 containing 15% FBS, 10 µM insulin, 0.5 µM isobutylmethylxanthine (IBMX, I5879, Sigma‐Aldrich), 1 µM dexamethasone (D4902, Sigma‐Aldrich), and 200 µM indomethacin (I8280, Sigma‐Aldrich) for 14 days. The study was approved by the research ethics committee of Xijing Hospital, Fourth Military Medical University, Xi'an, P. R. China and signed an informed consent by all donors (Permission Number: KY20194068).

##### Primary Culture and Differentiation of Mouse Brown Adipose‐Derived Stromal Cells

Brown adipose‐derived stromal cells were isolated and cultured as previously described.^[^ ^42^ ^]^ Briefly, BAT samples from 4 week old mice were minced and digested with collagenase solution (1 mg mL^−1^ collagenase II, 17101015, Thermo Fisher Scientific). Cells were then cultured in complete medium (DMEM/F12 medium containing 15% fetal bovine serum) for 24 h in a CO_2_ incubator (37 °C, 5% CO_2_). The medium was changed every 2 days. When cells grow to 100% confluence, medium was changed with induction medium (DMEM/F12 medium containing 15% FBS, 0.5 mM IBMX, 1 µM dexamethasone, 0.02 µM insulin (91077C, Sigma‐Aldrich), 1µM rosiglitazone (R2408, Sigma‐Aldrich), 1 nM T3 (T2877, Sigma‐Aldrich) in complete medium to induce the differentiation of brown adipose‐derived stromal cells. After incubation for 48 h, induction medium was changed with maintain medium (DMEM/F12 medium containing 15% FBS, 0.02 µM insulin, and 1 M n T3 in complete medium). The medium was changed every 2 days. On day 7–8, the cells were collected for experiments.

##### Cumulative Food Intake

Male mice were housed individually and provided with food (ND or HFD) and water ad libitum. The food consumption was measured at 10:00 a.m. every three days by recording the amount of chow remaining in the food dishes for 3 to 6 weeks.

##### Body Composition Measurement

Body composition was evaluated on anesthetized animals using live dual‐energy X‐ray absorptionmetry (InAlyzer, Medikors, Seungnam, South Korea).

##### Immunofluorescence, Immunohistochemistry, and Histological Analyses

Cells were fixed with 4% paraformaldehyde for 35 min, permeabilized with 0.2% Triton X‐100 for 10 min, and blocked with 2% bovine serum albumin (BSA) at room temperature for 60 min. After that, samples were incubated with primary antibody at 4 °C overnight and then incubated with the corresponding secondary antibody for 1 h at 37 °C. The following primary antibodies and dilutions were used: anti‐Flag (1:1000, Sigma‐Aldrich, China), anti‐ACSL1 (1:1000, Abclonal Technology, China), anti‐AKAP1 (1:1000, Abclonal Technology, China). Finally, samples were stained with diamidino‐2‐phenylindole (DAPI) at room temperature for 20 min. Images were captured using a laser scanning confocal microscope (TCS SP8, Leica, Germany). For Mitotracker staining, brown adipocytes were stained with MitoTracker Deep Red FM (Thermo Fisher Scientific) according to the manufacturer's instructions.

For hematoxylin–eosin (H&E) staining, mouse tissues were immediately fixed in 4% formaldehyde for 24 h and washed with 70% ethanol, embedded by paraffin, and continuously sectioned at 5 µM . The slides were imaged with Olympus microscope CX31. Then, adipocyte diameter measurement was performed with NIH ImageJ software (NIH, Bethesda, MD, USA) in at least three fields per slide (20× magnification). For immunohistochemistry, paraffin embedded tissue slides were deparaffinized and subjected to antigen retrieval in citric acid buffer, blocked with 5% normal goat serum, and incubated with antibodies: anti‐AKAP1 (1:300, Origene, China) or anti‐**UCP1** (1:100, Abcam, USA). Slides were then washed and incubated with the secondary antibody. Diaminobenzidine was used to visualize the target protein.

##### Oil Red O Staining

For Oil Red O staining, frozen liver sections were prepared in Tissue‐Tek OCT compound. Serial sections (8 µM were made and stained with 0.5% Oil Red O (O0625, Sigma‐Aldrich) for 30 min, counterstained with hematoxylin for 5 min. The red lipid droplets were visualized by microscopy (Olympus, CX31).

##### Biochemical Assay

Blood samples were obtained after an overnight fast. The level of blood glucose and plasma insulin was measured by glucometer (Roche, USA) and mouse insulin enzyme linked immunosorbent assay (ELISA) kit (90080, Crystal Chem, USA), respectively. Serum triglyceride, cholesterol, ALT, AST, BUN, and Cre levels were determined with an automatic biochemical analyzer (7170, Hitachi, Tokyo, Japan). The level of plasma NEFA was measured with a NEFA C Test Kit (#294‐63601, Wako Chemicals, Osaka, Japan). To test the liver triglyceride level, liver tissue was homogenized in a standard mix including chloroform/methanol (2:1). Subsequently, the organic phase in resolutions was dried down and redissolved in isopropanol. Liver triglyceride content was assessed using a Triglyceride Colorimetric Assay Kit (#MAK266‐1KT, Sigma‐Aldrich).

##### Glucose and Insulin Tolerance Tests

Glucose tolerance tests (GTTs) and insulin tolerance tests (ITTs) were monitored in awake mice that were fasted for 6 h (08:00–14:00) as described previously.^[^ ^43^, ^44^ ^]^ For intraperitoneal (i.p.) GTT, 24–28 and 14 week old HFD mice received an i.p. injection of 60 and 50 mg glucose (Sigma) per mouse, respectively. ND mice received an i.p. injection of 2 g glucose kg^−1^ of body weight. To calculate the baseline‐corrected area under the curve (AUC), basal glucose levels (time point 0) were subtracted from all later obtained blood glucose levels for each mouse individually. The AUC above the baseline glucose illustrated the glucose resistance in mice. For i.p. ITT, 24–28 and 14 week old HFD mice received an i.p. injection of 0.03 and 0.025 U insulin (Novo Nordisk) per mouse, respectively. ND mice received an i.p. injection of 0.75 U insulin kg^−1^ of body weight. Blood was taken by tail vein puncture and glucose levels were determined at the indicated time points using a glucometer (Roche, USA). To calculate the baseline‐corrected inverse AUC, basal glucose levels (time point 0) were subtracted from all later obtained blood glucose levels for each mouse. The values were inverted (multiplication with −1), followed by the calculation of the individual AUCs. Lower inverse AUC indicated the decreased insulin sensitivity.

##### Quantitative Real‐Time (qRT)‐Polymerase Chain Reaction (PCR) and Western Blotting

Total RNA from tissues or cells was extracted using RNAiso Plus (Takara, Japan). Total RNA (500 ng) was reverse transcribed to generate complementary DNA (cDNA) using PrimeScipt RT Reagent Kit with genomic DNA (gDNA) Eraser (Takara, Japan). qRT‐PCR was performed on CFX 96 Real‐Time System (Bio‐Rad, USA) using SYBR Premix Ex Taq II Kit (Takara, Japan) with specific primers, as listed in Table S1 (Supporting Information). Gene‐relative expression level was calculated using the ΔΔCT method. 36B4 and β‐actin were used as an internal control for adipose tissue and other tissues, respectively.

Snap‐frozen tissues, cells, and isolated mitochondria were lysed in radioimmunoprecipitation assay (RIPA) buffer supplemented with complete protease inhibitors (Roche), phenylmethylsulfonyl fluoride (PMSF), and phosphorylase inhibitor (Roche). Protein concentration was determined using the bicinchoninic acid (BCA) Assay Kit (Beyotime, Shanghai, China). Then, equal amounts of protein were separated by sodium dodecyl sulfate polyacrylamide gel electrophoresis (SDS/PAGE) gel and electrotransferred onto polyvinylidene fluoride (PVDF) membranes (Millipore). Then, the PVDF membrane was incubated with primary antibodies and horseradish peroxidase (HRP)‐conjugated secondary antibody. Subsequently, proteins bands were visualized by a chemiluminescence system (Amersham Bioscience, Buckinghamshire, UK) and quantified by ImageJ software (NIH, Bethesda, MD). All antibodies used in this study are listed in the section of Table S3 (Supporting Information).

##### Indirect Calorimetry Test

The metabolic parameters of mice were monitored using a Comprehensive Lab Animal Monitoring System (Columbus Instruments). Mice were acclimated for 24 h before measurements were taken. The oxygen consumption (VO_2_), exhaled carbon dioxide (VCO_2_), and physical activity of each mouse were determined and calculated for a 24 h period. The average values during light and dark period were calculated. Analysis of covariance (ANCOVA) was used to compare metabolic parameters of mice, in which body weight was used as covariate.

##### Body Temperature Measurement

For cold exposure experiments, mice were placed in a freezer (4 °C) with free access to food and water. The core body temperature was monitored at the indicated time points using a rectal probe (ThermoWorks, UT).

##### Infrared Thermography

The surface temperature of mice was recorded by an infrared thermography (FLIR Systems A310, USA). For cold‐induced thermogenesis, mice were placed at 4 °C and had free access to food and water. After 6 h, the surface temperature was recorded.

##### ^18^F‐FDG Micro‐PET Scanning

Whole‐body ^18^F‐FDG PET/computed tomography (CT) scanning was performed as described previously^[^ ^45^ ^]^ with an animal PET/CT scanner (Mediso Nano PET/CT, Hungary). Briefly, mice were fasted for 4 h with free access to water, and injected with 7.4 MBq (0.2 mCi) of ^18^F‐FDG via the tail vein. The imaging acquisition was started 30 min after tracer injection. For semiquantitative analysis, 3D regions of interest were carefully drawn and manually adjusted according to the CT images. The maximum standardized uptake values (SUV_Max_) were calculated based on the formula: SUV_Max_ = maximum tissue activity concentration (Bq mL^−1^)/injected dose (Bq) × body weight (g).^[^ ^46^ ^]^

##### Immunoprecipitation

Pierce Classic Magnetic IP/Co‐IP Kit (Thermo Fisher Scientific, USA) was used for AKAP1 immunoprecipitation (IP). Fresh BAT tissue or isolated BAT mitochondria was lysed with IP lysis buffer (Roche). The protein sample (500 µg) was immunoprecipitated with 10 µg anti‐AKAP1 antibody or anti‐ACSL1 antibody overnight at 4 °C. Subsequently, 20–40 µL of fully suspended protein A/G magnetic beads were added and incubated at room temperature for 1 h. The complexes that bound to the protein A/G conjugate were washed and resolved in the SDS–PAGE loading buffer, and subjected to Western blotting or liquid chromatography (LC)–MS/MS analysis.

##### LC–MS/MS Analysis of Proteins

After silver staining on the SDS–PAGE, the sliced gel samples were sent to Applied Shanghai Protein Technology Co. Ltd. (Shanghai, China) for LC–MS/MS analysis. The analysis was conducted on a Q Exactive mass spectrometer coupled to Easy nLC (Thermo Fisher Scientific, USA) using a routine method. MS data were acquired using a data‐dependent top10 method dynamically choosing the most abundant precursor ions from the survey scan (300–1800 m z^‐1^) for higher energy collisional dissociation (HCD) fragmentation. The data of MS were analyzed by MaxQuant software. Spectra data were searched against a UniProt mouse database (www.uniprot.org).

##### Chromatin Immunoprecipitation Assay

To validate the correlations between SP1 and AKAP1, SimpleChIP Plus Enzymatic Chromatin IP Kit (Magnetic Beads; #9005; Cell Signaling Technology) was used for ChIP assay according to the manufacturer's protocol with some modifications. In brief, 70 mg mouse BAT tissues were disaggregated into a single‐cell suspension after cross‐linking. The fragmented chromatins of the cells were immunoprecipitated with protein G magnetic beads and anti‐H3 (positive control), anti‐SP1 (active motif), or anti‐immunoglobulin G (anti‐IgG, negative control), then the immunoprecipitated chromatins were eluted. Finally, the recruited DNA was subjected to PCR analysis using the following primers: primer 1 (−153 to +75), forward, 5′‐GTCAGCGGAGAGCTCCTGAC‐3′, reverse, 5′‐GTGCTAGCCCAGGCCGGGCGAAC‐3′; primer 2 (−641 to −440), forward, 5′‐GGCAGTGACTCATGGTGACCATTTC‐3′, reverse, 5′‐CGTAACCTTGCGAGCATAGC‐3′. Mouse RPL30 primer was used for positive control in PCR reaction.

##### Isolation of Mitochondria

Mitochondria were isolated from BAT or mouse differentiated brown adipocytes using Minute Mitochondria Isolation Kit (Invent, MP‐007, USA) according to the manufacturer's protocol.

##### Mitochondrial ACS and PKA Enzymatic Activity Assays

The activity of acyl‐CoA synthetase (ACS) was measured by a continuous coupled enzymatic assay as previously described.^[^ ^47^ ^]^ The reaction mixture contained 100 mM Tris‐HCl buffer (pH 8.0), 10 mM ATP (10519979001, Sigma‐Aldrich), 15 mM MgCl_2_, 5 mM dithiothreitol, 150 mM KCl, 1 mM potassium phosphoenolpyruvate (P7127, Sigma‐Aldrich), 0.3 mM nicotinamide adenine dinucleotide (NADH, N8129, Sigma‐Aldrich) in 100 mM triethanolamine (pH 8.2), and 500 µM sodium palmitate (P9767, Sigma‐Aldrich). 4.5 µg adenylate kinase (M3003, Sigma‐Aldrich), 3 µg pyruvate kinase (P1506, Sigma‐Aldrich), 3 µg lactate dehydrogenase (L8080, Solarbio), and 3 µg mitochondrial protein sample were added in a total reaction volume of 100 µL. The reaction system was incubated at 37 °C for 1 min, and the reaction was initiated by the addition of CoA (final concentration 600 µM) (C4282, Sigma‐Aldrich). Changes in absorbance of the reaction system at 334 nm were measured every 10 s for 15 min with a recording spectrophotometer (Thermo Fisher Scientific). The reaction rate was calculated using the slope and intercept created from a NADH standard curve.

Total cellular and mitochondrial PKA activities were measured by the nonradioactive PKA Kinase Activity Assay Kit (ab139435, Abcam) following manufacturers' instructions.

##### Assessment of Oxygen Consumption Rate

Oxygen consumption rate (OCR) was assessed with a Seahorse XF24 extracellular flux analyzer (Agilent) as previously described.^[^ ^48^ ^]^ Brown preadipocytes from *AKAP1^−/−^* mice and WT mice were seeded at a density of 15 000 cells per well in a 24‐well Seahorse assay plate (Agilent) and differentiated as previously described. At day 7 of differentiation, 500 µM sodium palmitate (dissolved in BSA solution) was added. 24 h later, the cells were equilibrated for 0.5 h with Seahorse XF Roswell Park Memorial Institute (RPMI) medium (#103576‐100, Agilent), supplemented with 1.8 g L^−1^ NaCl, 1  mM pyruvate, 20 mM glucose, 2% BSA, and pen/strep. OCR was then measured under basal conditions and upon sequential injection of different reagents, including 3 µM oligomycin (75351, Sigma‐Aldrich), 1 µM norepinephrine (N‐069, Sigma‐Aldrich), 4 µM rotenone (R8875, Sigma‐Aldrich), and 4 µM antimycin A (A8674, Sigma‐Aldrich).

For isolated mitochondria (5 µg) of BAT, OCR was measured in a buffer containing 50 mM KCl, 4 mM KH_2_PO_4_, 5 mM 2‐[4‐(2‐hydroxyethyl)piperazin‐1‐yl] ethanesulfonic acid (HEPES), 1 mM ethylenebis (oxyethylenenitrilo) tetraacetic acid (EGTA), 2% fatty‐acid‐free BSA, 10 mM pyruvate (S8636, Sigma‐Aldrich), and 5 mM malate. Mitochondria were plated and centrifuged 2000 *g* for 20 min at 4 °C to enable the mitochondria to adhere to the plate. OCR was obtained under basal conditions and upon sequential injection of 1 µM oligomycin (Oligo), 1 µM carbonyl cyanide 4‐(trifluoromethoxy) phenylhydrazone (FCCP), 2 µM rotenone (R) and antimycin A (AA). The FCCP titration results were shown in Figure S3i (Supporting Information). For UCP1 function analysis, 1 mM guanosine diphosphate (GDP) was also added in the abovementioned buffer. After sodium palmitate titration analysis, 200 µM palmitate was selected to induce maximal OCR.

##### Determination of Fatty Acid Oxidation Rate

Fatty acid oxidation rates were determined as previously descripted.^[^ ^49^ ^]^ In brief, brown adipocytes were incubated in substrate‐limited medium overnight to initiate the adipocyte utilization of exogenous fatty acids. Before testing, the medium was replaced with FAO assay medium (111 mM NaCl, 4.7 mM KCl, 1.25 mM CaCl_2_, 2.0 mM MgSO_4_, 1.2 mM Na_2_HPO_4_, 2.5 mM glucose, 0.5 mM carnitine (C0283, Sigma‐Aldrich), and 5 mM HEPES) and incubated for 45 min. Then, palmitate–BSA (200 µM) was added into 24‐well Seahorse assay plate. Oligo 3 µM, FCCP (1 µM), R 4 µM, and AA 4 µM were injected sequentially. Etomoxir (40 µM) (E1905, Sigma‐Aldrich) was added 15 min before measurement to inhibit FAO.

##### LC–MS/MS Analysis of Palmitoyl Carnitine

Quantitative analysis of palmitoyl carnitine was carried out as previously descripted.^[^ ^50^ ^]^ The fresh BAT tissue or brown adipocytes were sent to Metware Biotechnology Co., Ltd. (Wuhan, China) for LC–MS/MS analysis. The sample extracts were analyzed using a liquid chromatography electrospray tandem mass spectrometry (LC–ESI–MS/MS) system (ultra performance liquid chromatography, UPLC, Shim‐pack; ultrafast liquid chromatography, UFLC, SHIMADZU CBM30A; MS/MS, Applied Biosystems 6500 QTRAP). Palmitoyl carnitine was analyzed using scheduled multiple reaction monitoring.

##### Adeno‐Associated Virus Infection

Recombinant adeno‐associated virus was constructed by GeneChem Co., Ltd. (Shanghai, China). AAVDJ vector expressing AKAP1 (AAVDJ–AKAP1) vector carrying mouse AKAP1 with an elongation factor 1α (EF1α) promoter was generated and AAVDJ empty vector (AAVDJ‐EV) was used as a negative control. To restore the expression of AKAP1 in BAT of *AKAP1^−/−^* mice, 6 week old *AKAP1^−/−^* mice were anesthetized by isoflurane. 50 µL AAVDJ–AKAP1–Flag (1 × 10^10^ vector genomes (VG) per mouse) or negative control AAVDJ–Flag was injected into interscapular BAT. For AAV infection in brown adipocytes, mouse brown adipose‐derived stromal cells were seeded at 4 × 10^5^ cells per well and infected 24 h later, with AAVDJ–AKAP1–Flag at a multiplicity of infection (MOI) of 2 VG per cell. The media were replaced 6 h postinfection. At day 7 of differentiation, cells were fixed for immunofluorescence staining.

##### Adenovirus Infection

Adenoviruses containing the sequences of mouse SP1 (*ad‐SP1*) were produced by Hanbio Biotechnology Co., Ltd. (Shanghai, China) for infection into brown adipocytes, and ad‐green fluorescent protein (ad‐GFP) was used as a negative control. The titer of adenovirus was 1.2 × 10^10^ plaque forming units (PFU) mL^−1^, and MOI was 100:1. After adenovirus infection and cell differentiation, the cells were subjected to PA for 24 h and harvested for further analysis.

##### siRNA Transfection

To knock down the expression of acyl‐CoA synthetase long chain family member 1 (ACSL1) in BAT of *AKAP1^−/−^* mice, *AKAP1^−/−^* HFD mice were anesthetized by isoflurane and were injected into interscapular BAT. 20 µg of ACSL1 siRNA or control siRNA was diluted in 40 µL vivo‐jetPEI and 10% glucose mixture and injected into the BAT. After 72 h of siRNA injection, BAT samples were collected for analysis of palmitoyl carnitine. For siRNA transfection in brown adipocytes, the culture plates were coated with gelatin. Then, siRNA and Lipofectamine RNAiMAX (Thermo Fisher Scientific) were diluted in Opti‐MEM and mixed. The final concentrations of Lipofectamine RNAiMAX and siRNA were 5 µL mL^−1^ and 50 nM, respectively. Then, the siRNA–RNAiMAX mix was added to gelatin‐coated culture plates. At day 5 of differentiation, mature brown adipocytes were digested and spun down (5 min, 300 g). The cells were resuspended in culture medium and added to gelatin‐coated culture plates on top of the preincubated siRNA–RNAiMAX mix. The mouse‐specific siRNAs targeting ACSL1 and SP1 were designed and synthesized by GenePharma (Shanghai) and the sequences of siRNAs were listed in Table S2 (Supporting Information).

##### Public Dataset Collection

Public microarray dataset including GSE92405, GSE59034, GSE77962, GSE48452, and GSE135066 were collected from GEO (www.ncbi.nlm.nih.gov/geo) for analysis of mRNA expression levels of AKAP1 and SP1 in different tissues under obesity condition. Dataset inclusion criteria were 1) a total of at least 20 samples; 2) no less than 5 cases and 5 controls; 3) gene expression profiling was available.

##### Peptide Synthesis and Injection

Peptides named AP‐30 and AP‐21 encompassed the 1–30 and 10–30 amino acids in mitochondrial targeting domain of AKAP1, respectively.^[^ ^51^ ^]^ A mutant AP‐21 peptide, which lost the ability to target mitochondria, was synthesized as a control in the in vivo experiments as previously described.^[^ ^20^ ^]^ Cell‐penetrating peptide (RRRRRR) was fused to the N‐terminal of peptides to enhance cellular uptake and drug delivery. The peptides were acetylated and amidated at N‐ and C‐terminal to reduce the helical dipole charge. The peptides AP‐30 (Ac—RRRRRRMAIQLRSLFPLALPGMLALLGWWWFFSRKK—NH_2_), AP‐21 (Ac—RRRRRRPLALPGMLALLGWWWFFSRKK—NH_2_), and mutant AP‐21 (Ac—RRRRRRPLALPGMLAAQGWAWFFSRKK—NH_2_) were synthesized according to the standard solid phase peptide synthesis by Ontores Biotechnologies Co. Ltd. (Hangzhou, China). In addition, fluorescein isothiocyanate (FITC) was coupled to the N terminus of peptides to determine the distribution and degradation rate of the peptides in the preliminarily investigation. Then, these peptides were purified via high performance liquid chromatography (95+%, pure), dissolved in saline, and subcutaneously injected into interscapular BAT area of mice.

##### Echocardiography

The detailed method for echocardiography is available in the Supporting Information.

##### Statistical Analysis

Statistical analyses were performed using GraphPad Prism 8.0 (GraphPad Software, Inc., La Jolla, CA). ANCOVA was performed using SPSS (IBM, Chicago, IL, USA). Data were expressed as mean ± standard error of the mean (SEM) and analyzed using Student's *t*‐test, one‐way or two‐way ANOVA followed by Bonferroni post hoc tests where appropriate. ANCOVA was used to compare metabolic parameters of the mice, in which body weight was used as the covariate. *p < 0.05, **p < 0.01.

## Conflict of Interest

The authors declare no conflict of interest.

## Supporting information

Supporting Information
