# Supplementary material for: AKAP1 Deficiency Attenuates Diet‐Induced Obesity and Insulin Resistance by Promoting Fatty Acid Oxidation and Thermogenesis in Brown Adipocytes
Source: Adv Sci (Weinh). 2021 Feb 1;8(6):2002794. doi: 10.1002/advs.202002794 (PMC7967052; doi:10.1002/advs.202002794)
Supplement: Supplementary file 1 — Supporting Information [file ADVS-8-2002794-s001.pdf]

WILEY-VCH

© 2020 Wiley-VCH GmbH

ADVANCED SCIENCE
Open Access

Supporting Information

for *Adv. Sci.,* DOI: 10.1002/advs.202002794

AKAP1 Deficiency Attenuates Diet-Induced Obesity and Insulin Resistance by Promoting Fatty Acid Oxidation and Thermogenesis in Brown Adipocytes

Lele Ji, Ya Zhao, Linjie He, Jing Zhao, Tian Gao, Fengzhou Liu, Bingchao Qi, Fei Kang, Gang Wang, Yilin Zhao, Haitao Guo, Yuanfang He, Fei Li, Qichao Huang, Jinliang Xing**

Supporting Information

AKAP1 Deficiency Attenuates Diet-Induced Obesity and Insulin Resistance by Promoting Fatty Acid Oxidation and Thermogenesis in Brown Adipocytes

Lele Ji[#], Ya Zhao[#], Linjie He[#], Jing Zhao, Tian Gao, Fengzhou Liu, Bingchao Qi, Fei Kang, Gang Wang, Yilin Zhao, Haitao Guo, Yuanfang He, Fei Li, Qichao Huang, Jinliang Xing**

Supporting Information includes:

Experimental Section

Figure S1-S8

Table S1-S3

Experimental Section

Generation of Genetically Modified Mice: *AKAP1*$^{-/-}$ mice on a C57BL6/N background were generated using CRISPR-Cas9 method. sgRNA (5'-CTTGGCGTTGCCCGGAATGC-3') was designed using the CRISPR tool (http://crispr.mit.edu) and synthesized by BGI (Beijing, China). Cas9 mRNA and sgRNA were microinjected into fertilized eggs. Mice carrying the mutant allele were backcrossed to C57BL6/N for 5 generations. Then the mice were intercrossed to generate *AKAP1*$^{-/-}$ mice. Genotypes were confirmed by Sanger sequencing (Songon Biotech, Shanghai, China). All animals used for experiments were bred by heterozygous mating. Considering the possible influence of estrogen on HFD-induced obesity in female mice, male *AKAP1*$^{-/-}$ mice were used in all experiments.

Echocardiography: Cardiac echocardiography were performed on lightly anesthetized mice using a Vevo 2100 echocardiography system (VisualSonics Inc., Toronto, Canada). In brief, cardiac function under physiological conditions was cursorily examined by hand-held manipulation of the ultrasound transducer to obtain two-dimensional and two-dimensional guided M-mode images. All echocardiographic parameters were analyzed using a Vevo 2100 software.

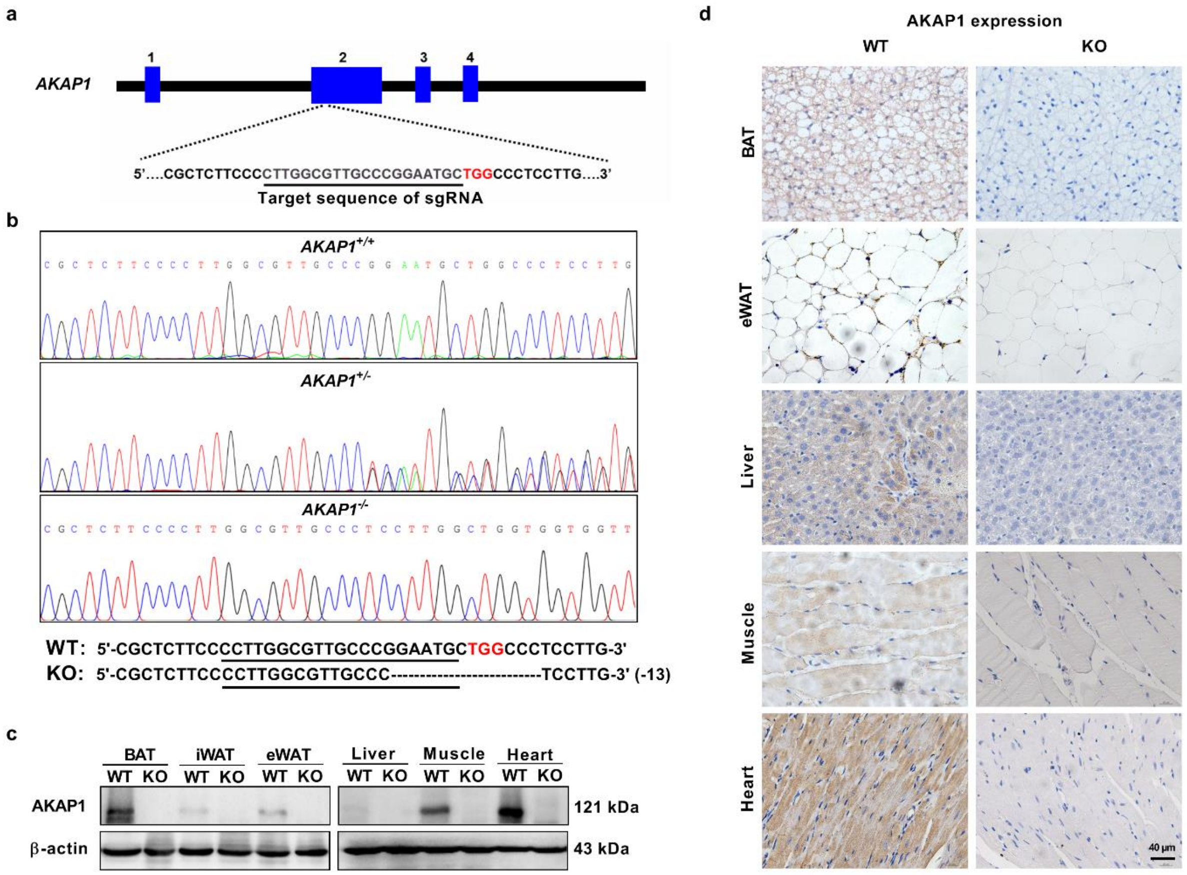

Figure S1. Generation of *AKAP1* knockout mice.

a) Schematic diagram of small guide RNA (sgRNA)-targeting site at Exon 2 locus of *AKAP1* gene.

b) Sanger sequencing of genomic DNA from wild type (WT) and knockout (KO) mice. The deletion of 13 base pairs indicated by hyphens resulted in a frame shift mutation.

c) Western blotting analysis of AKAP1 expression in different tissues from WT and KO mice. β-actin was used as a loading control.

d) Immunohistochemistry analysis of AKAP1 expression in different tissues from WT and KO mice. Scale bar, 40 μm.

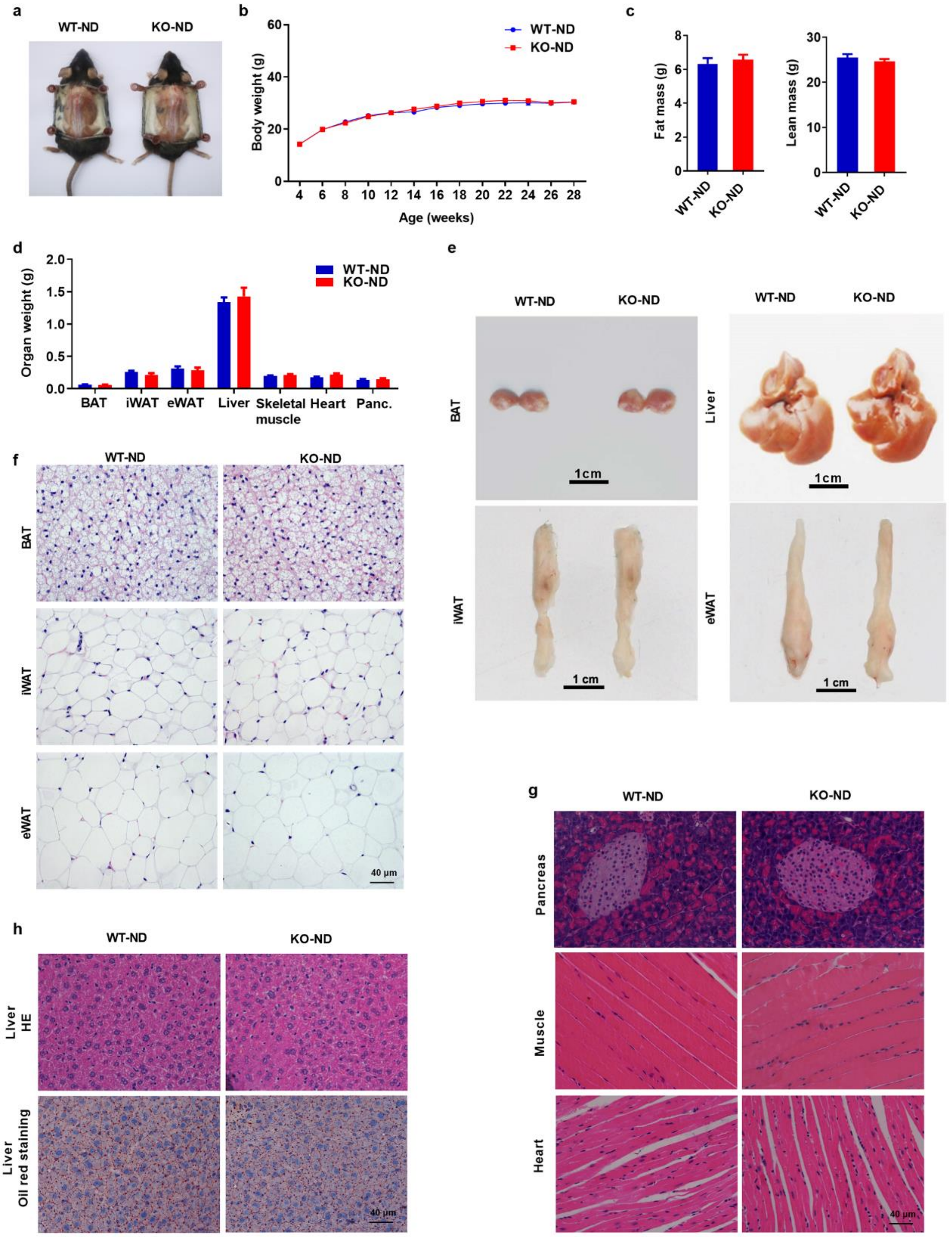

Figure S2. Body fat topography is comparable in wild type (WT) and AKAP1-deficient mice fed with a normal diet (ND).

a) Representative images of WT and *AKAP1*$^{-/-}$ mice on ND for 24 weeks.

b) Body weight of WT and *AKAP1*$^{-/-}$ mice on ND. WT-ND: Wildtype ND mice; KO-ND: knockout ND mice (n = 13 mice per group).

c) Fat and lean mass of WT and *AKAP1*$^{-/-}$ mice on ND for 24 weeks (n = 8 mice per group).

d) Organ weight of WT and *AKAP1*$^{-/-}$ mice on ND for 24 weeks (n = 8-10 mice per group).

e) Representative images of brown adipose tissue (BAT), inguinal white adipose tissue (iWAT), epididymal white adipose tissue (eWAT) and liver dissected from WT and *AKAP1*$^{-/-}$ mice on ND for 24 weeks. Scale bar, 1 cm.

f) Representative H&E-staining images of BAT, iWAT and eWAT from WT and *AKAP1*$^{-/-}$ mice on ND for 24 weeks. Scale bar, 40 μm.

g) Representative H&E-staining images of pancreas, muscle and heart from WT and *AKAP1*$^{-/-}$ mice on ND for 24 weeks. Scale bar, 40 μm.

h) Representative HE- and oil red-staining images of liver from WT and *AKAP1*$^{-/-}$ mice on ND for 24 weeks. Scale bar, 40 μm.

Data were expressed as mean ± SEM. Student's t test was used in c and d. Two-way ANOVA with Bonferroni's post hoc test was used in b. *$p < 0.05$, **$p < 0.01$.

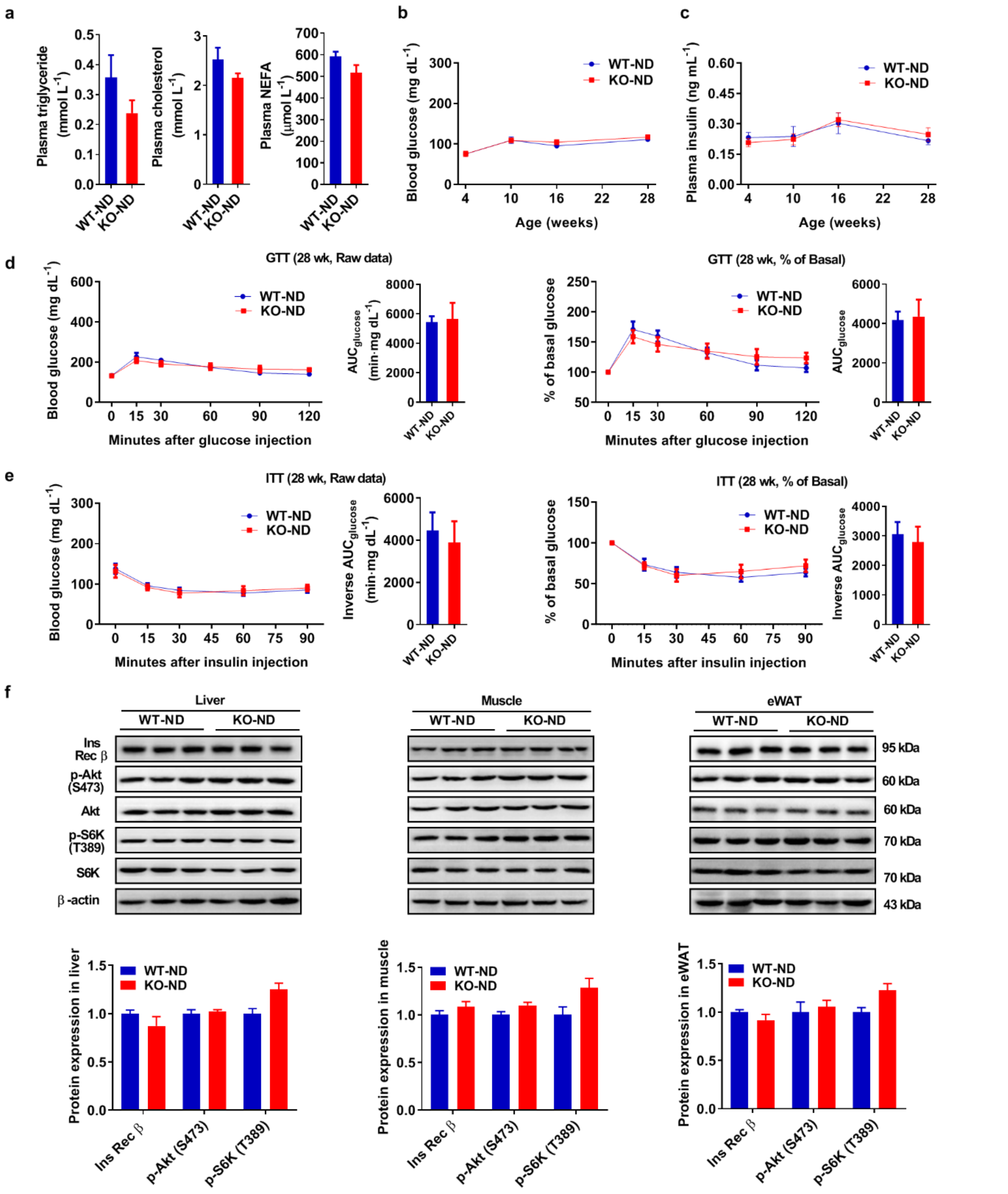

Figure S3. Abnormality in metabolic profile is not observed in AKAP-deficient mice on a normal diet.

a) Plasma triglyceride, cholesterol and nonesterified fatty acid (NEFA) levels of WT and *AKAP1*$^{-/-}$ mice on ND for 24 weeks (n = 6 mice per group).

b) and c) Blood glucose and plasma insulin level of WT and *AKAP1*$^{-/-}$ mice on HFD (n = 6

mice per group).

d) and e) Raw data of glucose tolerance test (GTT, n = 6 mice per group) and insulin tolerance test (ITT, n = 7-9 mice per group) of WT and *AKAP1*$^{-/-}$ ND mice by the age of 28 weeks (left). Blood glucose during GTT or ITT was expressed as a percentage of basal (Right). The area under the curve (AUC) was calculated for each mouse from both groups.

f) Western blotting analysis of Ins Rec β, p-Akt (S473), Akt, p-S6K (T389) and S6K expression in liver, muscle and eWAT from WT and *AKAP1*$^{-/-}$ mice on ND for 24 weeks which were sacrificed 15 min after i.p. injection of insulin (5 U kg^{-1}). n = 3 mice per group. β-actin was used as a loading control.

Data were expressed as mean ± SEM. Student's t test was used in a, d (AUC), e (AUC) and f. Two-way ANOVA with Bonferroni's post hoc test was used for analysis of the data in b, c, d (raw data of GTT) and e (raw data of ITT). *$p < 0.05$, **$p < 0.01$.

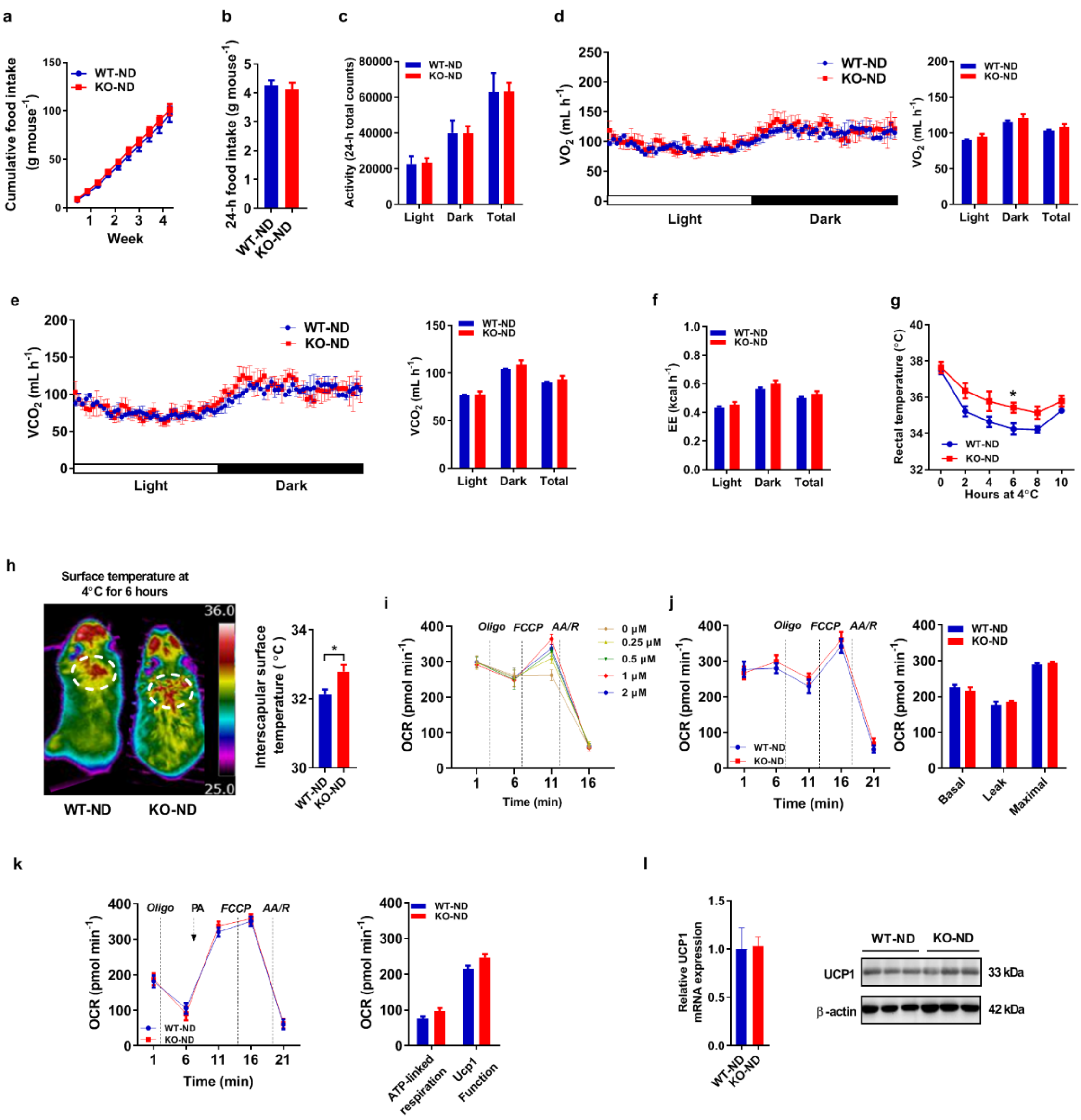

Figure S4. AKAP deficiency has no impact on energy expenditure in mice fed with a normal diet.

a) Cumulative food intake of WT and *AKAP1*$^{-/-}$ ND mice was measured every 3 days from 15 to 19 weeks of age (n = 5-6 mice per group).

b) 24 h Food intake and c) physical activity of WT and *AKAP1*$^{-/-}$ HFD mice (n = 4 mice per group).

d) and (e) Oxygen consumption (VO_2) and carbon dioxide expiration (VCO_2) during light and

dark phases were determined by metabolic cages in WT and *AKAP1*$^{-/-}$ ND mice (n = 4 mice per group).

f) Energy expenditure (EE) was estimated by the amount of O_2 consumption and CO_2 production in WT and *AKAP1*$^{-/-}$ ND mice (n = 4 mice per group).

g) Rectal core temperature of WT and *AKAP1*$^{-/-}$ ND mice exposed to 4℃ for 6 hours (n = 8 - 12 mice per group).

h) Representative infrared thermography of WT and *AKAP1*$^{-/-}$ ND mice exposed to 4°C for 6 hours (left). Surface temperature was quantified in the interscapular BAT area indicated by circle (right). n = 7 mice per group.

i) FCCP titration analyses for isolated BAT mitochondria from WT mice by seahorse analyzer. Mitochondria (5 µg) were incubated in buffer with the following compounds: 1 µM Oligomycin (Oligo), 0, 0.25, 0.5, 1 or 2 µM FCCP (F), and 4 µM each of rotenone (R) and antimycin A (AA).

j) Oxygen consumption rate (OCR) of BAT mitochondria was monitored by seahorse analyzer in WT and AKAP1$^{-/-}$ ND mice (n = 5 mice per group). Mitochondria (5 µg) were incubated in buffer with the following compounds: 1 µM Oligomycin (Oligo), 1 µM FCCP (F), and 2 µM each of rotenone (R) and antimycin A (AA). Basal OCR corresponds to baseline OCR minus AA/R-insensitive OCR; Leak OCR corresponds to Oligo-insensitive OCR minus AA/R-insensitive OCR; Maximal OCR corresponds to FCCP-induced OCR minus AA/R-insensitive OCR.

k) UCP1 function in BAT mitochondria of WT and AKAP1$^{-/-}$ ND mice (n = 5 mice per group) was monitored by seahorse analyzer. Respiration was measured with 1mM GDP, 10 mM pyruvate and 5 mM malate as substrates. Mitochondria (5 µg) were incubated in buffer with the following compounds: 1 µM Oligo, 1 µM FCCP (F), 200 µM palmitate and 4 µM each of

rotenone (R) and antimycin A (AA), respectively (n = 4 per group). ATP-linked respiration corresponds to baseline OCR minus Oligo-insensitive OCR; UCP1 function corresponds to palmitate addition phase OCR minus Oligo-insensitive OCR.

l) qRT-PCR and Western blotting analysis for UCP1 mRNA and protein expression in BAT from WT and *AKAP1*$^{-/-}$ ND mice (n = 3 mice per group).

Data were expressed as mean ± SEM. Student's t test was used in b, c, h, i, j and k. Two-way ANOVA with Bonferroni's post hoc test was used in a and g. The data in d, e and f were analyzed by analysis of covariance (ANCOVA) using body weight as the covariate. *$p < 0.05$, **$p < 0.01$.

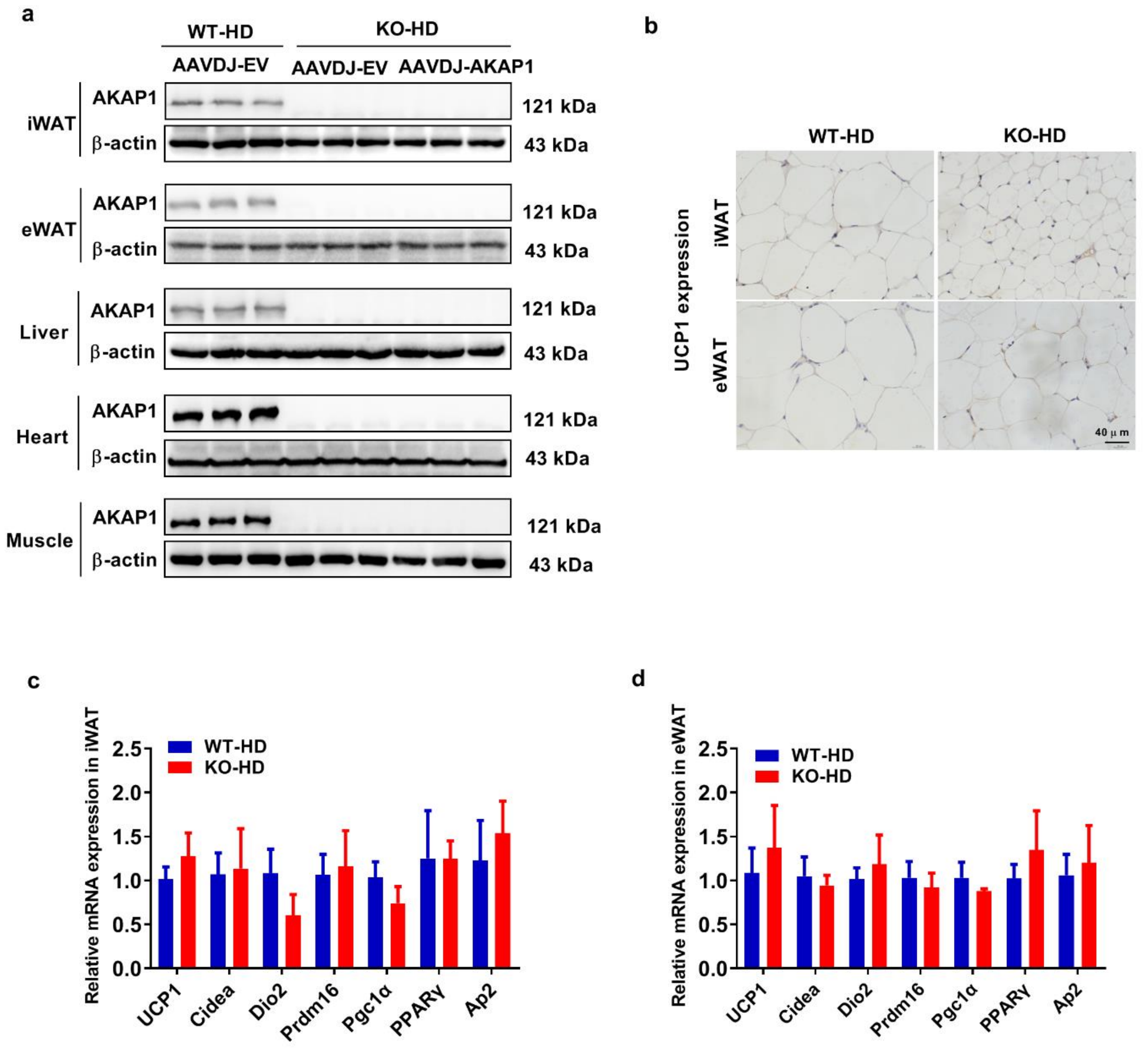

Figure S5. related to figure 4

a) Western blotting analysis of AKAP1 protein expression in other organs from WT and *AKAP1*$^{-/-}$ HFD mice injected with AAVDJ-EV or AAVDJ-AKAP1 (n = 3 mice per group). AAVDJ-EV, adeno-associated virus (AAVDJ) empty vector; AAVDJ-AKAP1: AAVDJ vector expressing AKAP1.

b) Representative immunohistochemistry-stained UCP1 sections of iWAT and eWAT in WT and *AKAP1*$^{-/-}$ HFD mice. Scale bar, 40 μm.

c) and d) Relative mRNA expression of AKAP1 expression in adipose tissues from WT and

AKAP1$^{-/-}$ HFD mice (n = 3 mice per group).

Data were expressed as mean ± SEM. Student's t test was used in c and d.

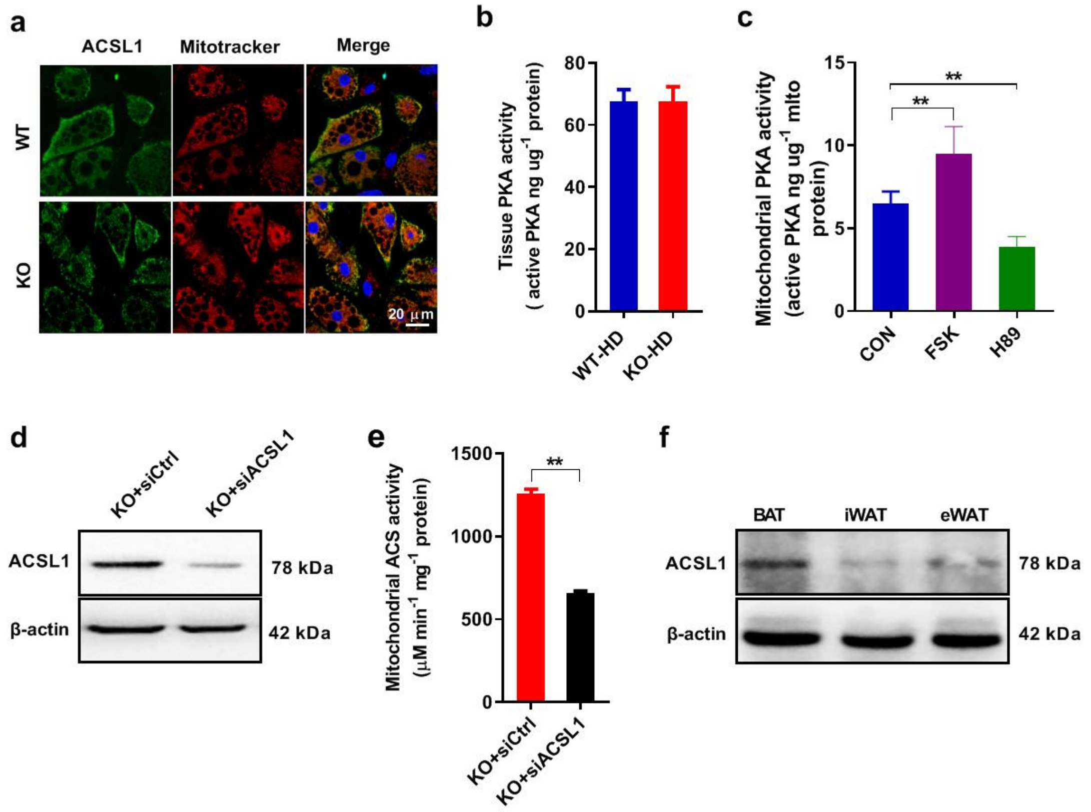

Figure S6. related to Figure 5.

a) Representative images of immunofluorescence for ACSL1 (green) and mitotracker (red) in brown adipocytes. Scale bar, 20 μm.

b) Total PKA activity in BAT from WT and *AKAP1*$^{-/-}$ mice on HFD (n = 6 mice per group).

c) Mitochondrial ACS activity in BAT from mice treated with PKA activator (FSK, 80μM) or PKA inhibitor (H89, 10 μM) (n = 6 mice per group).

d) Western blotting analysis of ACSL1 expression in *AKAP1*$^{-/-}$ brown adipocytes following siRNA transfection.

e) Mitochondrial ACS activity in *AKAP1*$^{-/-}$ brown adipocytes following siRNA transfection. Data are from three independent experiments.

f) Western blotting analysis of ACSL1 expression in mouse BAT, iWAT and eWAT..

Data were expressed as mean ± SEM. Student's t test was used in b and e. One-way ANOVA with Bonferroni's post hoc test was used in c.

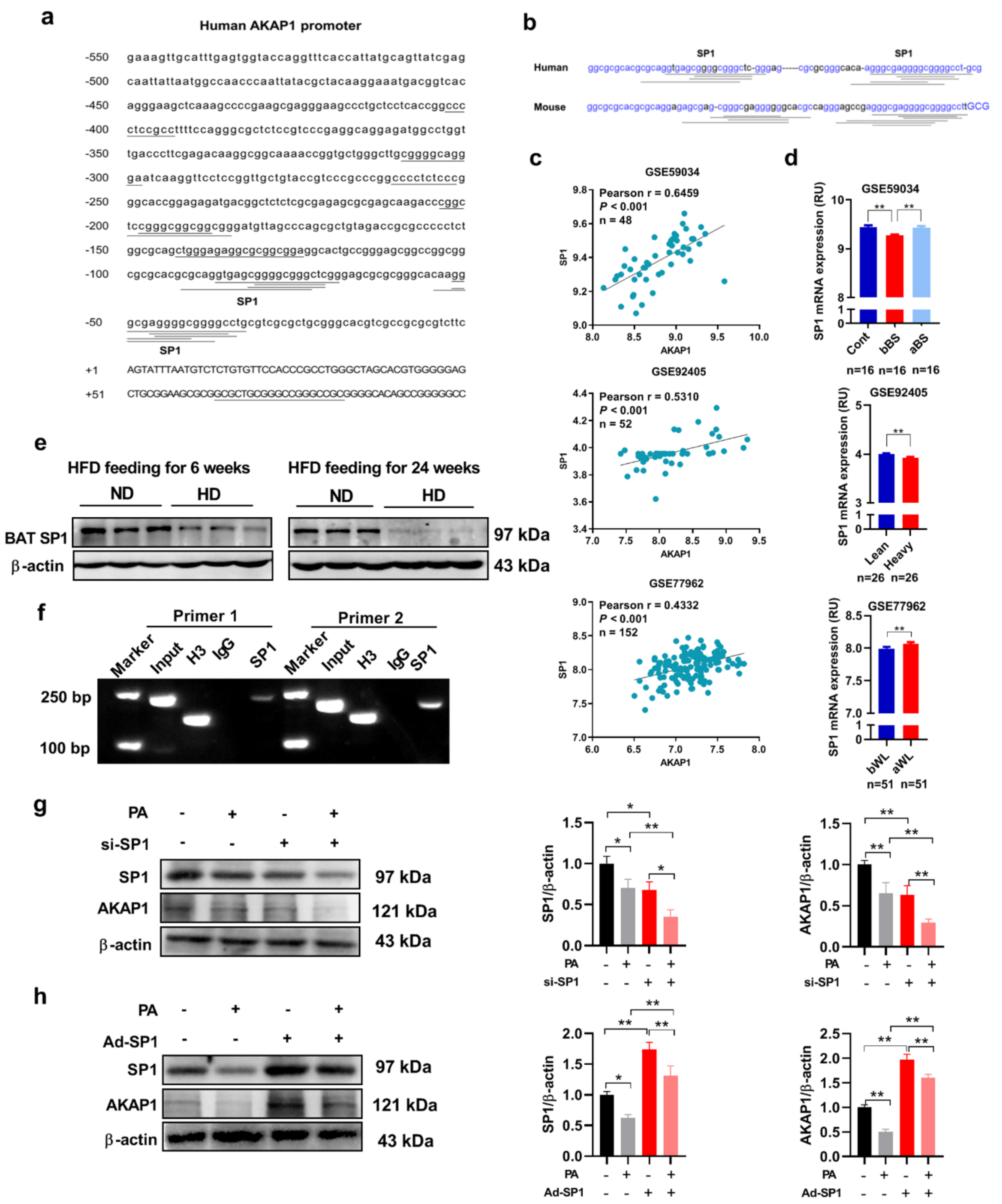

Figure S7. AKAP1 is downregulated by transcription factor SP1 in obesity.

a) Potential binding sites for the transcription factor SP1 in the human AKAP1 promoter region.

b) Conserved putative Sp1 binding sites in the promoter region of AKAP1 gene in human and mouse.

c) Pearson's correlation analysis between SP1 and AKAP1 mRNA expression in human subcutaneous adipose tissue based on GEO datasets (GSE59034, GSE92405, GSE77962). Pearson r values are shown.

d) The mRNA expression analysis of SP1 in human subcutaneous adipose tissues in GEO datasets (GSE59034, GSE92405, GSE77962). Cont, never-obese control; bBS, before bariatric surgery; aBS, after bariatric surgery; bWL, before weight loss (at study start); aWL, after weight loss period.

e) Western blotting analysis of SP1 protein expression in BAT of HFD mice (n = 3 mice per group).

f) ChIP analysis of SP1 binding to the AKAP1 promoter in mouse BAT.

g) and h) Quantification of Western blotting analysis for SP1 and AKAP1 expression in differentiated mouse brown adipocytes with treatment as indicated (palmitate, PA, 500 μM, 24 h). Data are representative of three independent experiments.

Data were expressed as mean ± SEM. Student's t test was used in d (GSE92405, GSE77962). One-way ANOVA with Bonferroni's post hoc test was used in a (GSE59034). Correlations between measured variables were tested using Pearson correlation analysis in c. *$p < 0.05$, **$p < 0.01$.

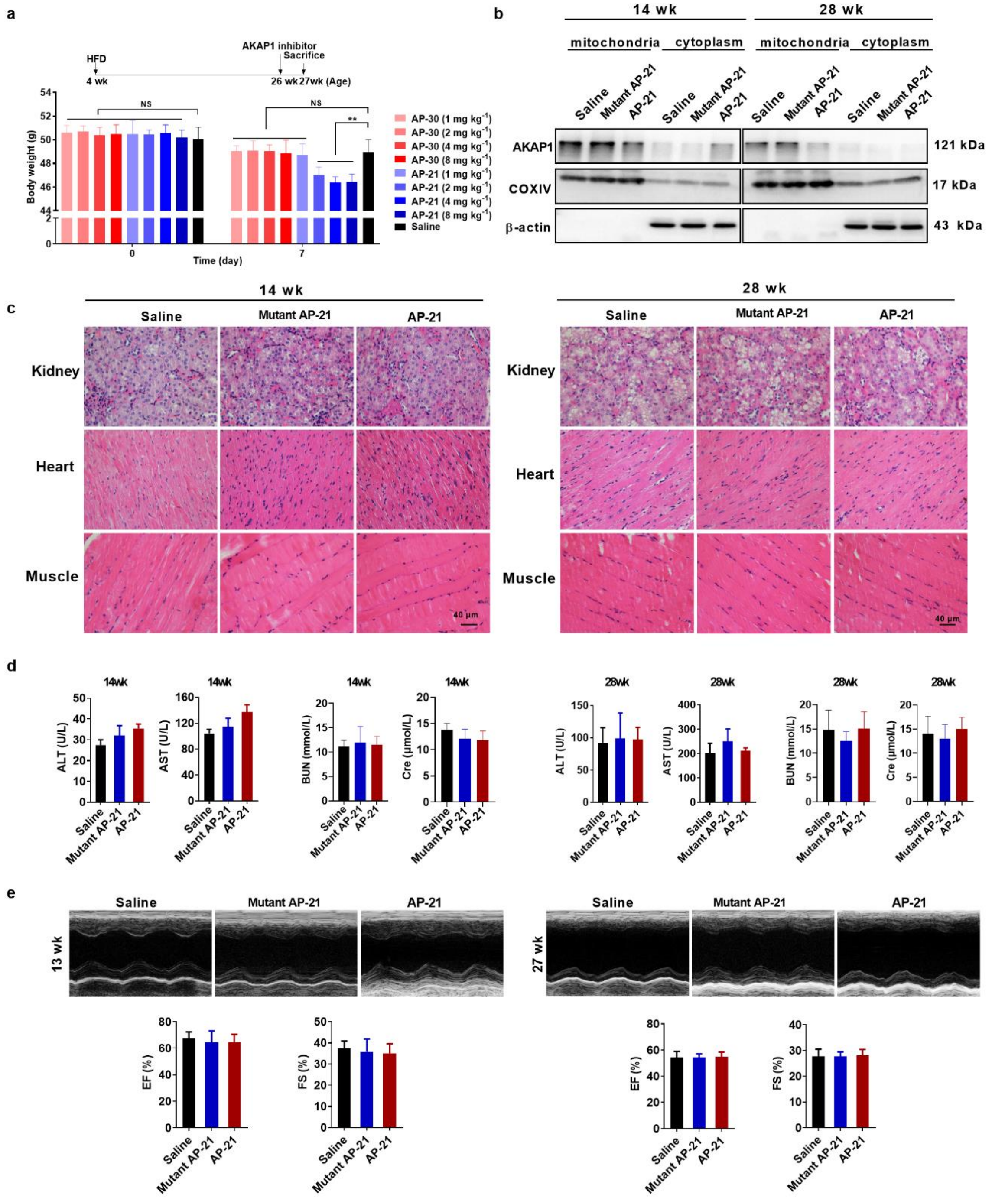

Figure S8. AKAP1 inhibitor treatment has no detrimental impact on cardiac function in mice.

a) Body weight of HFD-treated mice injected with AKAP1 inhibitor AP-21 or AP-30 (n = 4 mice per group).

b) Western blotting analysis of AKAP1 expression in mitochondria or cytoplasm of BAT from mice with AP-21 treatment (n = 3 mice per group). COXIV and β-actin were used as loading

controls.

c) Representative H&E-staining images of kidney, heart and muscle from HFD mice with treatment as indicated. Scale bar, 40 μm.

d) The levels of serum alanine aminotransferase (ALT), aspartate aminotransferase (AST), blood urea nitrogen (BUN) and serum creatinine (Cre), of HFD mice with treatment as indicated. (n = 8-12 mice per group for 14-wk-old HFD mice; n = 5-7 mice per group for 28-wk-old HFD mice).

e) Ejection fraction (EF) and fractional shortening (FS) of HFD mice with treatment as indicated (n = 3-4 mice per group).

Data were expressed as mean ± SEM. Two-way ANOVA with Bonferroni's post hoc test was used in a. One-way ANOVA with Bonferroni's post hoc test was used in d and e. $*p < 0.05$, $**p < 0.01$

Table S1. The sequence of Primers

Gene	Forward (5'-3')	Reverse (5'-3')
AKAP1 (Mouse)	GTTGCCCGGAATGCTGG	TGGGGAGGTTGCCTGAGGA
36b4 (Mouse)	GCAGACAACGTGGGCTCCAAGCAGAT	GGTCCTCCTTGGTGAACACGAAGCCC
UCP1 (Mouse)	GGCATTCAGAGGCAAATCAGCT	CAATGAACACTGCCACACCTC
Cidea (Mouse)	TGCTCTTCTGTATCGCCCAGT	GCCGTGTTAAGGAATCTGCTG
Pgc1a (Mouse)	AATACCGCAAAGAGCACGAG	ACCAACGTAAATCACACGGC
Ap2 (Mouse)	ACACCGAGATTTCCTTCAAACTG	CCATCTAGGGTTATGATGCTCTTCA
Adiponectin (Mouse)	GCACTGGCAAGTTCTACTGCAA	GTAGGTGAAGAGAACGGCCTTGT
Dio2 (Mouse)	CAGTGTGGTGCACGTCTCCAATC	TGAACCAAAGTTGACCACCAG
Prdm16 (Mouse)	CAGCACGGTGAAGCCATTC	GCGTGCATCCGCTTGTG
PPARγ (Mouse)	TGTCGGTTTCAGAAGTGCCTTG	TTCAGCTGGTCGATATCACTGGAG
β-actin (Mouse)	AGCCATGTACGTAGCCATCCA	TCTCCGGAGTCCATCACAATG
AKAP1 (Human)	AGCCGAGGAGCTGGTGTAAT	GGGAGAGGTTCCTTGATGGC
36b4 (Human)	TCGTGGAAGTGACATCGTCTTT	CTGTCTTCCCTGGGCATCA

Table S2. siRNA sequence of Primers

Target	siRNA sequence (5'-3')
ACSL1	GGAUGCUUCUCUUACUCAA
SP1	GGAUGCUUCUCUUACUCAA
Negative	UUCUCCGAACGUGUCACGUTT

Table S3. Primary antibodies used for Western blotting, immunoprecipitation, immunofluorescence and immunohistochemistry

**Antibody**	**Company**	**Catalogue Number**
Custom-made rabbit polyclonal anti-AKAP1 (WB, IF, IP)	Abclonal Technology	
Rabbit Anti-AKAP1 (IHC)	ORIGENE	Cat# TA324184
Rabbit monoclonal anti-Akt (pan) (C67E7)	Cell Signaling Technology	Cat# 4691; RRID:
Rabbit monoclonal anti-Phospho-Akt (Ser473)	Cell Signaling Technology	Cat# 4060; RRID: AB_2315049
Rabbit monoclonal anti-Insulin Receptor β (4B8) mAb	Cell Signaling Technology	Cat# 3025; RRID:
Rabbit monoclonal anti-Phospho-p70 S6 Kinase (Thr389)	Cell Signaling Technology	Cat# 9202S; RRID: AB_2285392
Rabbit monoclonal anti-p70 S6 Kinase (49D7)	Cell Signaling Technology	Cat# 2708S; RRID: AB_390722
Phospho-(Ser/Thr) PKA Substrate Antibody	Cell Signaling Technology	Cat# #9621; RRID: AB_330304
Rabbit polyclonal anti-COX IV	Cell Signaling Technology	Cat# 4844; RRID: AB_2085427
Mouse monoclonal anti-Flag	Sigma-Aldrich	Cat# F1804; RRID: AB_262044
Rabbit polyclonal anti-IgG	Sigma-Aldrich	Cat# 12-370; RRID: AB_145841
Rabbit polyclonal anti-UCP1(IHC)	Abcam	Cat# ab10983; RRID: AB_2241462
Rabbit polyclonal anti-UCP1(WB)	proteintech	Cat# 23673-1-AP; RRID: AB_2828003
Rabbit polyclonal anti-ACSL1 (WB, IF)	proteintech	Cat# 13989-1-AP; RRID: AB_2257870
Rabbit polyclonal anti-ACSL1 (IP)	Abclonal Technology	Cat# A1000; RRID: AB_2757519
Rabbit polyclonal anti-VDAC1	proteintech	Cat# 10866-1-AP; RRID: AB_2257153
Rabbit polyclonal anti-SP1 (CHIP)	Active Motif	Cat# 39058; RRID:AB_2793151
Goat anti-Rabbit IgG (H+L) Secondary Antibody, HRP	TDY Biotech	Cat# S004
Goat anti-Mouse IgG (H+L) Secondary Antibody, HRP	TDY Biotech	Cat# S001
Rabbit polyclonal anti-β-actin	TDY Biotech	Cat# TDY051
